# Natural Products for Pancreatic Cancer Treatment: From Traditional Medicine to Modern Drug Discovery

**DOI:** 10.3390/nu13113801

**Published:** 2021-10-26

**Authors:** Ahyeon Kim, Jiwon Ha, Jeongeun Kim, Yongmin Cho, Jimyung Ahn, Chunhoo Cheon, Sung-Hoon Kim, Seong-Gyu Ko, Bonglee Kim

**Affiliations:** 1 College of Korean Medicine, Kyung Hee University, Seoul 02447, Korea; ahyeon8022@khu.ac.kr (A.K.); jiwooonha@khu.ac.kr (J.H.); kje654@khu.ac.kr (J.K.); 2 Department of Pathology, College of Korean Medicine, Kyung Hee University, Seoul 02447, Korea; ymcho@khu.ac.kr (Y.C.); skyajm911@khu.ac.kr (J.A.); sungkim7@khu.ac.kr (S.-H.K.); 3 Korean Medicine-Based Drug Repositioning Cancer Research Center, College of Korean Medicine, Kyung Hee University, Seoul 02447, Korea; hreedom@khu.ac.kr (C.C.); epiko@khu.ac.kr (S.-G.K.)

**Keywords:** pancreatic cancer, natural product, traditional medicine, apoptosis, angiogenesis, metastasis, drug resistance

## Abstract

Pancreatic cancer, the seventh most lethal cancer around the world, is considered complicated cancer due to poor prognosis and difficulty in treatment. Despite all the conventional treatments, including surgical therapy and chemotherapy, the mortality rate is still high. Therefore, the possibility of using natural products for pancreatic cancer is increasing. In this study, 68 natural products that have anti-pancreatic cancer effects reported within five years were reviewed. The mechanisms of anti-cancer effects were divided into four types: apoptosis, anti-metastasis, anti-angiogenesis, and anti-resistance. Most of the studies were conducted for natural products that induce apoptosis in pancreatic cancer. Among them, plant extracts such as *Eucalyptus microcorys* account for the major portion. Some natural products, including *Moringa*, Coix seed, etc., showed multi-functional properties. Natural products could be beneficial candidates for treating pancreatic cancer.

## 1. Introduction

Pancreatic cancer is a disease in which malignant cancer cells develop in the tissue of the pancreas. Due to concealed clinical manifestation, limited treatment options, and side effects, pancreatic cancer is considered one of the most difficult cancers to treat. Jaundice, belly or back pain, poor appetite, and weight loss are typical signs and symptoms of pancreatic cancer [1]. The cure rate for this disease is only 9%, and if not treated, the median survival of patients with metastatic disease is only about three months worldwide [2]. According to GLOBOCAN 2018, 458,918 cases were newly diagnosed, and 432,242 deaths were reported worldwide in 2018. These rates account for 2.5% and 4.5% of all cancer cases globally in 2018. Pancreatic cancer was estimated to be the seventh most common cancer in both men and women. Meanwhile, pancreatic cancer was more common in developed countries, including Europe, North America, East Asia, and Australia [3].

Surgical resection is well known as the most effective treatment for pancreatic cancer. Because there are many cases in which surgical resection is not available due to late presentation, chemotherapy is often used as adjuvant treatment [4]. FOLFIRINOX and gemcitabine/albumin-bound nab-paclitaxel are considered the first-line treatment against pancreatic cancer. For patients with BRCA1/2 and PALB2 mutations, gemcitabine/cisplatin is a suitable treatment. Because the selection of an available treatment depends on a number of factors, including patient preference, comorbidities, goals of treatment, and predictive biomarkers, treatment is not quite so simple in fact [2]. In addition, the risk of recurrence and the possibility of side effects remain. Therefore, using only existing drugs is not enough for covering pancreatic cancer, and it is essential to develop some new drugs.

Traditional medicine around the world, including China, Japan, Thailand, India, and Korea, is drawing attention these days. Traditional Chinese medicine (TCM) has been widely used in China using treatments accumulated over thousands of years. Thus, TCM occupies an important position throughout traditional medicine. Traditional Thai medicine (TTM) is a Buddhism-based health care system in Thailand that includes herbal medicine, massage, midwifery, etc. [5]. Ayurveda medicine in India, which emphasizes ‘balance’, has been with Indians in their daily lives for more than 5000 years [6]. Traditional Korean medicine (TKM) is a unique medicine that has developed independently for 5000 years [7]. TKM has established its own medical identity, through *Euibangyoochui*, *Donguibogam* compiled by Jun Heo and Sasang constitutional medicine established by Je-ma Lee [8]. Treatments of TKM such as acupuncture, moxibustion, and herbal medicine are still widely used today. TKM attracted worldwide attention with the growth of complementary and alternative medicine (CAM). In particular, natural product-based herbal medicine is currently expected to be a novel treatment of several diseases including cancer, due to its effectiveness and lack of serious side effects [9]. 

Anticancer effects of natural products are being proved through experimental studies in various types of cancers, such as lung, breast, colon, and prostate cancer. Several natural compounds, including curcumin, resveratrol, berberine, baicalein, dioscin, wogonin, piperine, etc., were reported to have an anti-cancer effect [10,11,12,13,14,15,16]. In addition, natural product-derived compounds are known to induce apoptosis in cancer cells rather than in normal cells [17]. Thus, natural products will play a key role as a novel cancer treatment for the next decade.

Most representative anti-cancer mechanisms include: apoptosis, anti-metastasis, anti-angiogenesis, resistance, etc. Apoptosis or programmed cell death (PCD) is a prime cellular mechanism to control cell proliferation and remove harmful or unnecessary cells from an organism [18]. Apoptosis can be regulated by targeting Bcl-2 family members and caspases. Meanwhile, some defects in the process of apoptosis can lead to tumor metastasis and resistance [19]. Metastasis means that malignant cancer cells spread from primary tumors to other sites, thereby resisting treatment and causing organ dysfunction [20]. Anti-angiogenesis is a process that inhibits novel blood vessels formed in pre-existing ones. Resistance is a mechanism that decreases the effect of anticancer drugs, typically driven by irreversible genetic mutations [21]. 

Therefore, we aim to review the experimental studies about natural products against pancreatic cancer, analyzing original research in terms of apoptosis, anti-metastasis, anti-angiogenesis, and resistance. Only studies published in the last five years were included in this paper. Several natural compounds which were combined with radiotherapy or enhanced the anticancer activity of gemcitabine are also reviewed. Furthermore, our present study included clinical trials which were conducted to evaluate the efficacy and safety of natural products when treating pancreatic cancer.

## 2. Apoptosis Inducing Natural Products

Apoptosis, known as programmed cell death is regarded as a significant component of numerous processes including defense mechanisms like immune responses, when cells are damaged by disease or toxic agents [22]. Inadequate apoptosis, too much or too little, can lead to a variety of diseases, including many types of cancer such as pancreatic cancer. Apoptosis is one of the major target mechanisms when treating cancer. It has been observed that a wide variety of natural products trigger apoptosis, but they do not affect the cell lines to die in the same mechanism.

### 2.1. Apoptosis Inducing Fungi

Five natural products from fungi were reported to have an apoptotic effect on pancreatic cancer cells (Table 1). 

*Agaricus blazei* Murrill is the most frequently used medicinal mushroom in Japan. Matsushita et al. showed that its water extract (AbE) induced cell cycle arrest and increased nuclear fragmentation [22]. Cleavages of caspase-3, -9, and PARP1 indicate that AbE induces apoptosis via caspase-dependent pathway. In addition, overexpression of the genes which encode proapoptotic proteins, such as DEDD2, DAPK3, and NLRP1, was observed after AbE treatment. 

Chaetospirolactone is a natural product that is isolated from the endophytic fun-gus *Chaetomium* sp. NF00754 [23]. Both in vitro and in vivo, chaetospirolactone induced apoptosis without interrupting the normal pancreatic cells of HPDE6c-7 cell line. Chaetospirolactone treatment sensitized AsPC-1 and PANC-1 cells, which are TRAIL (Tumor necrosis factor-related apoptosis inducing ligand)-resistant cells. As a result, TRAIL-mediated apoptosis occurred in a dose-dependent manner, and cleaved bands of caspase-8, -9, and -3 were detected. 

Dicatenarin and Skyrin, secondary metabolites from fungus *Penicillium pinophilum*, induced apoptosis via reactive oxygen species (ROS)-mediated mitochondrial pathway in MIA PaCa-2 cells [24]. The activation of cytochrome c and caspase-3 and the loss of MTP were observed. Because of an additional phenolic hydroxyl group at C-4, dicatenarin could generate more ROS. Thus, dicatenarin is slightly more effective than skyrin as a pancreatic cancer treatment. 

Both Xylarione A and (-) 5-methylmellein, which were isolated from fungus *Xylaria psidii*, induced cell cycle arrest and led to apoptosis [25]. Hence, 10, 30, and 50 µm of these compounds were treated for 24 h in MIA PaCa-2 cells. As a result, the MMP (mitochondrial membrane potential, ΔΨM) loss was observed, indicating that these compounds triggered apoptosis through mitochondrial damage.

### 2.2. Apoptosis Inducing Marine Sponge

One compound from the marine sponge was reported to have an apoptotic effect on pancreatic cancer cells (Table 2). Leiodermatolide, isolated from a marine sponge *Leiodermatium*, was treated to identify apoptosis in AsPC-1, BxPC-3, MIA PaCa-2, and PANC-1 cells [27]. In this study, cleavage of caspase-3 was most remarkable after 24 h of Leiodermatolide treatment in BxPC-3 and MIA PaCa-2 cells. In an orthotopic xenograft mouse model of pancreatic cancer, reduction of tumor weight was successful. However, the survival rate was not significantly increased. 

### 2.3. Apoptosis Inducing Plants

Forty-three plant extracts and their compounds were reported to have apoptotic effects on pancreatic cancer cells (Table 3). 

#### 2.3.1. Natural Compounds from Plants

In a study, *Andrographis paniculata* 70% EtOH extracts showed 21 known compounds [28]. Lee et al. demonstrated that 14-deoxy-11,12-didehydroandrographolide (compound 17) had the strongest preferential toxicity against PANC-1 and PSN-1 cell lines. When the cell lines were treated with compound 17, apoptosis-like cell death appeared in a time- and dose-dependent manner. 

The compound 2′,4′-Dihydroxy-6′-methoxy-3′,5′-dimethylchalcone (DMC) originated from *Cleistocalyx operculatus* [29]. When PANC-1 cells were exposed to 3, 10, and 30 μM of DMC for 48 h, activation of Bax, cytochrome c, c-caspase-3, -9, and c-PARP, and reduction of Bcl-2 were observed. 

In addition, 5,7-dihydroxy-3,6,8-trimethoxyflavone (flavone A), extracted from *Gnaphalium* *elegans*, triggered apoptosis through the mitochondrial intrinsic pathway in PANC-28 cells which are relatively differentiated pancreatic cancer cells [30]. Meanwhile, 3,5-dihydroxy-6,7,8-trimethoxyflavone (flavone B), extracted from *Achyrocline bogotensis*, induced apoptosis through the extrinsic pathway in Mia-PaCa-2 cells which are relatively poorly differentiated pancreatic cancer cells. 

Zhang et al. demonstrated that 8-Chrysoeriol mainly targets and inhibits Bcl-2, showing cytotoxicity against SW1990 cells overexpressed with Bcl-2 [31]. After SW1990 cells were exposed to 50 and 100 μM of 8-Chrysoeriol for 24 h, the rate of apoptotic cell death increased. Notably, at 100 μM, the rate surged to 79.8%. 

Tian et al. reported that various cardiac glycosides, derived from seeds of *Thevetia peruviana*, had inhibitory effects on three cancer cell lines, namely P15, MGC-803, and SW1990, and one normal hepatocyte cell, LO2 [32]. Cardiac glycosides also turned out to have a selective inhibitory effect on three tumor cells. 

Crocetinic acid, extracted from *Crocus sativus*, inhibited the proliferation of pancreatic cancer cells [33]. Fraction 5 had the strongest anti-cancer effect both in vitro and in vivo among five fractions isolated from commercial crocetin. In vitro, five fractions were treated to MiaPaCa-2 cells. In vivo, 6–8-weeks old athymic female mice bearing MiaPaCa-2 cells were treated with 0.5 mg/kg crocetinic acid for 21 days. Crocetinic acid up-regulated c-caspase 3 and Bax, and down-regulated PCNA, p-EGFR, p-AKT, and Bcl2, leading to apoptosis. 

As a kind of steroid sapogenin, diosgenin is derived from *Solanum*, *Dioscorea*, and *Costus* species [34]. Diosgenin induced apoptotic cell death and cell cycle arrest in Patu8988 and PANC-1 cells. EZH2, which is known to be an oncogenic protein of several cancers, and its target vimentin was down-regulated in pancreatic cancer cells after diosgenin treatment. 

Echinacoside (ECH), which is isolated from stems of *Cistanchessalsa*, induced apoptosis by elevating ROS and reducing MMP in SW1990 cells [35]. ROS elevation and MMP decrease have been reported to be necessary for the induction of apoptosis. Moreover, Wang et al. investigated that ECH upregulated the expression of Bax, which is triggered by the tumor suppressor p53. Further, ECH triggers apoptosis via mitogen-activated protein kinase (MAPK) pathway, suppressing JNK and ERK1/2 activity, while enhancing p38 activity. However, ECH did not affect AKT activity, which is also an important mechanism in cell proliferation. 

Elemene, extracted from *Zingiberaceae* plants, inhibited cell proliferation and induced cell cycle arrest in a dose-dependent manner in BxPC-3 and PANC-1 cells [36]. Elemene treatment also had an apoptotic effect on in vivo model. In this study, up-regulation of p53 (tumor suppressor gene) and down-regulation of Bcl-2 (apoptosis-related gene) in BxPC-3 bearing BALB/c nude mice model were observed by Western blot method. 

MicroRNAs (miRNA) are small non-coding RNA molecules which function in the post-transcriptional regulation of gene expression, and their functions are related to mRNA molecules [37]. Wang et al. demonstrated that grape seed proanthocyanidins (GSPs), an active component of *Vitis vinifera* (grape) seed, inhibited the growth in PANC-1 by modulating miRNA expression. GSPs down-regulated miRNA-SS3, SS12, and SS24, and also down-regulated CDK6, EGFR, MSH6, and DNMT1. This indicated that the negative co-expression correlations between DE miRNAs and target genes showed that GSPs may play an anti-cancer role by regulating miRNAs’ expression. 

Guha et al. demonstrated that hydroxychavicol induces apoptosis through JNK-dependent and caspase-mediated pathway [38]. The expression of c-caspase-3, -8, -9, c-Bid, c-PARP, and Bax increased, while the expression of Bcl-2 was suppressed in MIA PaCa-2 and PANC-1 cells after hydroxychavicol treatment. Notably, the activation of caspase-8 and -9 indicates that both extrinsic and intrinsic apoptotic pathways were induced in the hydroxychavicol-treated model. 

Hyperoside and hypoxoside, which are natural prodrugs, induced caspase-dependent apoptosis against MIA PaCa-2 and INS-1 pancreatic cancer cells [39]. Activation of cleaved capase-3, a key factor of apoptosis, was remarkable in this study. These compounds also caused G2/M cell cycle arrest and a hydrolyzed form of hyperoside and hypoxoside showed selective cytotoxicity. 

Icariin, purified from traditional chinese medicine *Herba Epimedii*, induced apoptosis and inhibited migration and proliferation in PANC-2 cells [40]. This study also included an in vivo experiment, treating 120 mg/kg of icariin in C57BL/6 mice for 11 days. The result showed that icariin affects the tumor immune microenvironment, thus inhibiting pancreatic cancer growth. 

Isothiocyanates, major compounds of cruciferous vegetables, have been widely known to have cancer prevention effects [41]. Luo et al. showed that Methyl4-(2-isothiocyanatoethyl) benzoate (compound 6) and N-Ethyl-4-(2-isothiocyanatoethyl) benzamide (compound 7) especially had a more apoptotic effect in PANC-1 but were less noxious to non-cancer cells. Compound 7 up-regulated ROS, and down-regulated GSH, resulting in apoptosis of pancreatic cancer cells. 

Mastic gum resin (MGR), isolated from *Pistacia atlantica* subspecies kurdica, at concentrations from 0.01 to 100 μM for 72 h enhanced apoptosis in PANC-1 [42]. Rahman demonstrated that the resin had the anti-proliferative effect not only in pancreatic cancer cells, but also in bile duct cancer, gastric adenocarcinoma, and colonic cancer. 

Monogalactosyl diacylglycerol (MGDG), extracted from a spinach, induced apoptosis in cancer cells when it is used alone or with radiation [43]. The combination of MGDG and radiation resulted in a higher percentage of apoptosis than each single treatment, and also inhibited tumor growth in a mouse xenograft model. Meanwhile, up-regulated gene expression of cytochrome c, c-PARP, c-caspase-3, and Bax and down-regulation of Bcl-2 were observed in MIA PaCa-2 cells when treated with MGDG. 

Wang et al. reported that piperlongumine, originated from the fruit of the pepper *Piper longum*, up-regulated the level of procaspase-3 and c-PARP in BxPC-3, PANC-1, and AsPC-1 cells [44]. In addition to in vitro experiments, in vivo model also showed anticancer activity against pancreatic cancer. In BxPC-3 bearing BALB/c mice treated with 10 mg/kg of piperlongumine for 21 days, tumor growth was significantly inhibited. When piperlongumine is combined with gemcitabine in vitro and In vivo, an apoptotic effect was enhanced. The expression of Bcl-2, Bcl-xL, survivin, XIAP, c-Myc, cyclin D1, COX-2, VEGF, and matrix metalloproteinase-9 (MMP-9), all of which are regulated by NF-κB, was decreased after combination treatment. 

Karki et al. demonstrated that piperlongumine, an anti-cancer natural compound found in *Piper longum*, induced apoptosis and inhibited cell growth by inducing ROS in PANC-1 with 5 to 15 μmol/mL treatment [45]. In PANC-1 cells, a piperlongumine-induced ROS decrease triggered a down-regulation of miR-27a, miR-20a, and miR-17, which are miRNAs regulated by cMyc. Moreover, this results in the downregulation of Sp1, Sp3, and Sp4. These mechanisms are critically related to the action of piperlongumine against pancreatic cancer cells. L3.6pL bearing athymic nu/nu mice were treated with piperlongumine in the amount of 30 mg/kg for 21 days. Accompanying down-regulation of Sp1, Sp3, and Sp4, piperlongumin induced inhibition of tumor weight, without affecting body weight. Cotreatment with glutathione was effective both in vitro and in vivo. 

Zhang et al. demonstrated that RN1, a polysaccharide from the flower of *Panax notoginseng*, inhibited PDAC cell growth both in vitro and In vivo, which is highly related to Gal-3 [46]. RN1 had a dose-dependent apoptotic effect on AsPC-1 and BxPC-3 cells, while having no effect on other cancer cells such as L-02 (normal liver cell line) and HPDE6-C7 (normal pancreatic cell line). RN1 specifically inhibited Gal-3 expression by binding to Gal-3, which inhibited the activation of the EGFR/ERK signaling pathway as well. Gal-3 expression was also inhibited by Runx1 In vitro. RN1 inhibited Gal-3 expression and down-regulated EGFR/ERK/Runx1 signaling In vivo. Thus, RN1 works as a novel Gal-3 inhibitor and is expected to be a potent anti-pancreatic cancer cell treatment via multiple mechanisms and pathways. 

Rottlerin, which is isolated from *Mallotus philippinensis*, triggered cellular apoptosis in Patu8988 and PANC-1 cells [47]. An increase of cytochrome c release was observed. This compound also induced cell cycle arrest, inhibited cell proliferation, and delayed cell migration and invasion. When Skp2 (S-phase kinase associated protein 2) is overexpressed, cancer growth could not be suppressed effectively. In this study, the inactivation of Skp2 was significant after rottlerin treatment, indicating that rottlerin could be a potential agent for pancreatic cancer therapy. 

Sugiol, a kind of diterpene, showed antiproliferative activity and induced apoptosis in Mia-PaCa2 cells [48]. Bax elevation and Bcl-2 depression suggest that sugiol triggered apoptosis through mitochondrial pathway. When the concentration of sugiol increased, intracellular ROS was up-regulated, whereas MMP level was down-regulated. 

Several anticancer activities of Withaferin A (WA, a steroidal lactone isolated from *Withania somnifera*) and Carnosol (CA; an ortho-diphenolic diterpene included in rosemary, sage, and oregano) were demonstrated in this study [49]. WA and CA induced early apoptosis in AsPC-1 cells. However, late apoptosis and necrosis were insignificant. In terms of the HGF-mediated c-Met signaling pathway, phosphorylation of c-Met and Akt was attenuated. WA and CA also had an inhibitory effect on cell proliferation, cell migration, and cell cycle arrest. 

Li et al. investigated whether WA suppresses proteasome activity and induces ER stress [50]. This WA-induced ER stress resulted in cellular apoptosis, and up-regulation of c-caspase-3, -8, -9, c-PARP1 was observed in PANC-1 and MIA PaCa-2 cells. Meanwhile, when WA is combined with a series of ER stress aggravators, c-PARP level elevates, and cell viability decreases, suggesting that the treatment enhanced apoptotic effect.

#### 2.3.2. Plants Extracts

Bitter apricot ethanolic extract (BAEE) originated from *Prunus armeniaca* L. led to apoptosis through a mitochondrial-dependent pathway [51]. Therefore, the Bax/Bcl-2 ratio and level of caspase-3 were both increased in PANC-1 cells. To determine cell cytotoxicity, apoptosis, and necrosis, PANC-1 (human pancreatic cancer cells) and 293/KDR (normal epithelial cells) were used in this study. All cellular apoptosis occurred without affecting normal epithelial cells. 

Non-polar stem extracts (SN) of *Clinacanthus nutans* were effective for inducing apoptosis in AsPC-1, BxPC-3, and SW1990 cells [52]. This compound up-regulated the level of Bax and down-regulated the level of Bcl-2, cIAP-2, and XIAP. The synergistic effect occurred when SN extracts were combined with gemcitabine, thereby improving anti-pancreatic cancer activity compared to those treated respectively. 

Qian et al. showed that In vitro, pre-treatment with coix seed emulsion (CSE) significantly up-regulated caspase-3, c-PARP, and Bax, leading to the synergetic effect of gemcitabine in three types of pancreatic cancer cell lines: BxPC-3, PANC-1, and AsPC-1 [53]. Moreover, the pre-treatment down-regulated anti-apoptotic substances like Bcl-2, survivin, and COX-2. In vivo, co-treatment of coix seed emulsion and gemcitabine had a more potent apoptotic effect than using them separately. Six-week old male nude BALB/c mice bearing human BxPC-3 cells were treated with 12.5 mL/kg CSE for 24 days. This resulted in a decrease in p65. Conclusively, despite the CSE dose-dependently induced apoptotic effect in pancreatic cancer cell lines, the combination of CSE and gemcitabine turned out to be potent in pancreatic cancer than using them separately. 

Cordifoliketones A is a compound extracted from Tsoong, the roots of *Codonopsis cordifolioidea* [54]. Luan et al. showed that treatment with cordifoliketones A inhibited growth and induced apoptosis of AsPC-1, BxPC-3, and PANC-1 both in vitro and in vivo. In vitro, treatment of 2, 4, and 6 µg/mL cordifoliketones A to three types of PDAC cells for 24 and 48 h turned out to have an apoptotic effect. Moreover, 6 µg/mL cordifoliketones A-treated groups showed stronger apoptosis compared to the other groups which were treated with different doses. Additionally, cordifoliketones A did not affect normal human cells. In vivo, BALB/c nude mice bearing human AsPC-1, BxPC-3, and PANC-1 cells alone (placebo) and mice bearing the same PDAC cells with the treatment with cordifoliketones A (control) were compared. It was proven that the control had a slower PDAC proliferation rate than the placebo. 

Bhuyan et al. reported that *Eucalyptus microcorys* leaf aqueous extract had an anti-proliferative effect against pancreatic cancer cells [55]. F1, which is one of the five major fractions of the extract, had an especially prominent apoptotic effect against MIA PaCa-2. F1 up-regulated Bak, Bax, c-PARP, and c-caspase-3, and down-regulated Bcl-2, procaspase-3, which led to apoptosis. Moreover, gemcitabine and F1 showed a synergistic apoptotic effect when combined. 

Another study by Bhuyan et al. demonstrated that selected Eucalyptus species inhibited the growth of various cancer cells including lung and pancreatic cancer cells by more than 80% [56]. Aqueous and ethanolic *Eucalyptus microcorys* leaf extract, ethanolic *Eucalyptus microcorys* fruit extract, and *Eucalyptus saligna* ethanolic extract had an apoptotic effect in MIA PaCa-2. MIA PaCa-2, treated with 100 μg/mL aqueous *Eucalyptus microcorys* leaf and fruit extract for 24 h, showed significant apoptosis compared to other extracts. Growth inhibition was much stronger when MIA PaCa-2 was treated with 100 μg/mL than 50 μg/mL. The selected Eucalyptus species had a great apoptotic effect in MIA PaCa-2 compared to BxPC-3 and CFPAC-1. 

Pak et al. showed that the herbal mixture ethanol extract (H3) from *Meliae Fructus*, the bark of *Cinnamomum cassia*, and *Sparganium rhizome* had an antitumor effect in vitro and in vivo in PANC-1 cells [57]. H3 inhibited proliferation of PANC-1, induced apoptosis, induced G0/G1 cell cycle arrest, and down-regulated apoptosis-related mRNAs like CXCR4, JAK2, and XIAP. Moreover, the anticancer activity of H3 was confirmed by up-regulation of cytochrome c and down-regulation of COX-2. In vivo, five-week old BALB/c nude mice bearing human PANC-1 cells were divided into four groups: control, treated with H3, gemcitabine, and H3+gemcitabine. The group treated with only H3 showed significant necrotic cell death and RBC-containing cavities in tumor tissue. H3 up-regulated cytochrome c and down-regulated COX-2 In vivo. 

Ethyl acetate extract of *Inula helenium* L. (EEIHL) inhibited the proliferation of pancreatic cancer cells and activated apoptosis in CFPAC-1 cells [58]. EEIHL treatment up-regulated mRNA level of E-cadherin, and down-regulated mRNA level of Snail, which leads to cell adhesion. Additionally, EEIHL down-regulated the phosphorylation of STAT3 and AKT. A low concentration of EEIHL induced cell arrest and high concentration of EEIHL enhanced apoptosis by the phosphorylation mechanism. 

Aqueous leaf extract of *Moringa oleifera* has been reported to exhibit anticancer activity [59]. When this compound is combined with radiation, the expression of PARP-1, Bcl-2, COX-2, and p65 protein is down-regulated, increasing the inhibitory effect on tumor growth. In vivo, PANC-1 bearing mice treated with 1.5 mg/g *moringa* showed the smallest tumor volume compared with control, 0.5 mg/g, and 1.0 mg/g groups, suggesting that *moringa* suppressed tumor growth in a dose-dependent manner. 

The root bark of *Paeonia suffruticosa* is known to inhibit the growth of cancer and metastasis [60]. Liu et al. reported that treatment of *P. suffruticosa* aqueous extracts (PS), aqueous extract from *Paeonia suffruticosa*, augmented caspase-3, -8, and -9, showing the activation of apoptosis. PS-elevated ROS and accumulated ER stress lead to inhibition of autophagy and proteasomes, which triggers apoptosis of PANC1, AsPC-1, and BxPC-3 cells. 

*Pterospermum acerifolium* ethanolic bark extract induced apoptosis and cytotoxic effect in PANC-1 cells by up-regulating mitochondrial-mediated ROS production [61]. *P. acerifolium* bark extract also showed apoptosis against lung cancer cells (A549). The extract up-regulated ROS generation and arrested both types of cancer cells by inducing early apoptosis of cells before the G1 phase. Additionally, PANC-1 cells were more sensitive to *P. acerifolium* bark extract than A549. 

Polyphenol-rich extract of *Salvia chinensis* is reported to exhibit an inhibitory effect on breast, lung, and colon cancer cells [62]. This compound also induced cell cycle arrest and apoptosis in MIA PaCa-2 cells. The increase of cytochrome c release and the loss of MMP suggest that this compound leads to a mitochondrial apoptotic pathway. 

The extract of *Sedum sarmentosum Bunge* (SSBE), originated from traditional Chinese herbal medicine, is known to have antiviral, anticancer, and anti-inflammatory properties [63]. This compound activated the expression of caspase-3, -8, Bax, and Bad, and suppressed the level of Bcl-2 in PANC-1 cells at a dose of 100 µg/mL. SSBE-treated PANC-1 cells also showed a p53 increase and a c-Myc decrease. In animal xenograft models of pancreatic cancer, tumor weight was reduced by inhibition of Hedgehog signaling after SSBE treatment. 

Total flavonoid aglycones extract (TFAE), derived from *Radix Scutellariae*, has been previously reported to suppress lung cancer [64]. Liu et al. showed that TFAE also had anti-cancer activity against pancreatic cancer [65]. After TFAE treatment, cleavages of caspase-3, -9, PARP, and Bid increased whereas Bax and Bcl-2 were unchanged in BxPC-3 cells. Inhibition of TFAE-induced autophagy increases apoptosis, suggesting that autophagy and apoptosis are in a cross-regulatory relationship. Meanwhile, the mice with 150 mg/kg TFAE had the strongest inhibitory effect against tumor growth, compared with the mice which were treated with lower doses.

#### 2.3.3. Formulations

F35, isolated from *Inula helenium* L., is a mixture of alloalantolactone, alantolacton, isoalantolactone in a ratio of 1:5:4 [66]. Its anti-cancer activity was investigated in two types of pancreatic cancer cells: PANC-1, SW1990, and was compared with isoalantolactone’s anti-tumor activity. Even though isoalantolactone is the main component from *Inula helenium* L., it is hard to gain pure isoalantolactone by current methods. Fortunately, F35, which is relatively easy to extract, strongly inhibited the growth of PANC-1 and SW1990 with a treatment of 8 µg/mL of F35 at 48 h. In addition, when treated with 6 µg/mL of F35 for 24 h, both groups of cells showed mitochondrial-dependent apoptotic activity, just as they were treated with isoalantolactone. Treating PANC-1 and SW1990 with 2 µg/mL of F35 for 24 h eliminated colony formation and with 2 or 4 µg/mL of F35 for 24 h inhibited migration.

**Figure 1:**
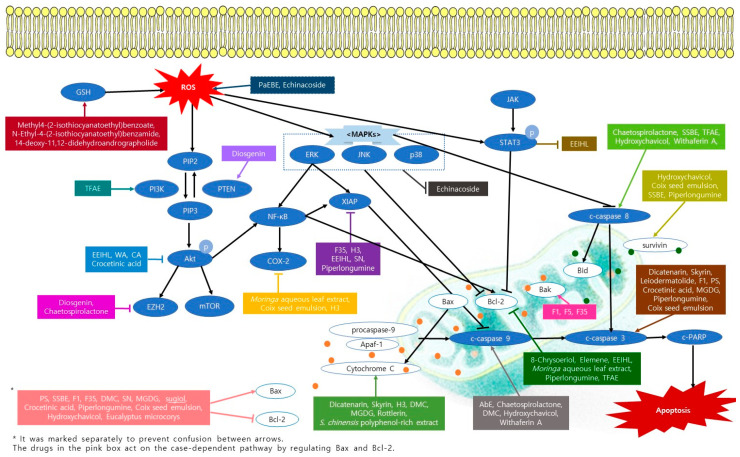
Schematic diagram of apoptotic mechanisms of various natural products against pancreatic cancer. The intrinsic apoptotic pathway is regulated primarily by anti-apoptotic Bcl-2 family of proteins. Bcl-2 inhibits release of mitochondrial cytochrome c. Cytochrome c which is released from the mitochondrial intermembrane forms the apoptosome complex in the cytosol with Apaf-1 and procaspase-9, leading to caspase-9 activation. Caspase-9 then activates effector caspases, resulting in some cleavages of cellular proteins and finally cell death by apoptosis. Several natural products such as 8-Chrysoeriol, Elemene, EEIHL, *Moringa* aqueous leaf extract, Piperlongumine, TFAE, PS, SSBE, F1, F35, DMC, SN, MGDG, sugiol, Crocetinic acid, Coix seed emulsion, Hydroxychavicol, and *Eucalyptus microcorys* down-regulated Bcl-2. Dicatenarin, Skyrin, H3, DMC, MGDG, Rottlerin, S. chinensis polyphenol-rich extract induced cytochrome c release to the cytosol. Some drugs affected on caspase-3, 8, and 9 straight without going through the process. Meanwhile, MAPKs signaling pathway, stimulated by ROS, could also inhibit anti-apoptotic Bcl-2 proteins. In addition to MAPKs, several processes triggered by ROS act directly or indirectly on apoptosis.

Apoptosis, also called programmed cell death, is a well-known mechanism for treating various cancers, including pancreatic cancer. Forty-nine substances were reported to induce apoptosis in pancreatic cancer. The apoptotic mechanisms of natural products were illustrated in Figure 1. Most of the substances belonged to the plant, except for five fungus and one marine sponge. The pancreatic cancer cell lines commonly used for apoptosis were MIA PaCa-2, PANC-1, AsPC-1, and BxPC-3. In vivo, BALB/c mice were mainly used as an animal model. As a result, the rate of apoptotic cell death increased after treatment, mostly in a dose- and time-dependent manner. 

Chaetospirolactone, xylarione A, (-) 5-methylmellein, 2′,4′-Dihydroxy-6′-methoxy-3′,5′-dimethylchalcone (DMC), 3,5-dihydroxy-6,7,8-trimethoxyflavone (flavone B), 5,7-dihydroxy-3,6,8-trimethoxyflavone (Flavone A), cardiac glycosides, elemene, hydroxychavicol, hypoxoside, mastic gum resin (MGR), Methyl4-(2-isothiocyanatoethyl)benzoate (compound 6), monogalactosyl diacylglycerol, N-Ethyl-4-(2-isothiocyanatoethyl)benzamide (compound 7), piperlongumin, RN1, withaferin A, bitter apricot ethanolic extract, *Eucalyptus microcorys* aqueous extract (F1), and *Salvia chinensis* polyphenol-rich extrac, showed almost no cytotoxicity in normal cells while having an apoptotic effect on pancreatic cancer cells [24,26,29,30,32,36,38,39,41,42,43,44,46,49,51,55,62]. The rest of the studies on the apoptosis of pancreatic cells did not handle experiments on normal cells, so additional tests are required. However, the researchers pointed out that additional in vivo experiment is necessary to ensure the safety and effectiveness of these substances. 

Seventeen substances, namely Chaetospirolactone, leiodermatolide, cordifoliketones A, diosgenin, elemene, icariin, monogalactosyl diacylglycerol, piperlongumin, RN1, withaferin A, coix seed emulsion, herbal mixture extract (H3), *Moringa* aqueous leaf extract, *Paeonia suffruticosa* aqueous extracts (PS), *Sedum sarmentosum Bunge* extract, and total flavonoid aglycones extract, were investigated for their anti-cancer effects in mice models [24,27,34,36,40,43,44,45,46,50,51,53,54,57,59,60,63,65]. The rest of the studies had only in vitro experiments. Some studies suggested that clinical trials with natural compounds are needed. Meanwhile, because leiodermatolide and monogalactosyl diacylglycerol showed toxicity when treated with high doses, a further investigation with low doses experiment was suggested [27,43]. 

**Table 3:** Apoptosis inducing plants.

Classification	Compound/Extract	Source	Cell Line/Animal Model	Dose; Duration	Efficacy	Mechanism	Reference
Plant	14-deoxy-11,12-didehydroandrographolide	*Andrographis paniculata*	PANC-1, PSN-1	6.25, 12.5, 25, 50, 100 µM; 12, 16, 20, 24 h	Induction of apoptosis	↓GSH	[28]
Plant	2′,4′-Dihydroxy-6′-methoxy-3′,5′-dimethylchalcone (DMC)	*Cleistocalyx operculatus* buds	PANC-1	3, 10, 30 μM; 48 h	Induction of apoptosis	↑Bax, cytochrome c, c-caspase-3, -9, c-PARP ↓Bcl-2	[29]
Plant	3,5-dihydroxy-6,7,8-trimethoxyflavone (flavone B)	*Achyrocline bogotensis*	Mia PaCa-2	40 μM; 6 h	Induction of apoptosis and cell cycle arrest	↑p-ERK, p-c-JUN ↓pS6	[30]
	5,7-dihydroxy-3,6,8-trimethoxyflavone (flavone A)	*Gnaphalium elegans*	PANC-28	40 μM; 6 h		↓p-ERK, pS6, p-Bad, Bcl-xL, Bcl-2	
Plant	8-Chrysoeriol		SW1990	50, 100 μM; 24 h	Induction of apoptosis	↓Bcl-2	[31]
Plant	Cardiac glycosides	seed of *Thevetia peruviana*	SW1990		Inhibition of proliferation of tumor cell lines		[32]
Plant	Carnosol	*Rosmarinus officinali,* *Salvia carnosa,* *Origanum vulgare*	AsPC-1	1 μM; 48 h	Induction of apoptosis		[49]
Plant	Crocetinic acid	*Crocus sativus*	MIA PaCa-2	1, 10, 25, 50 µM; 72 h	Induction of apoptosis Inhibition of proliferation	↑c-caspase 3, Bax ↓CD133, DCLK-1, Shh, PCNA, p-EGFR, p-AKTa, Bcl-2	[33]
			MIA PaCa-2 bearing athymic nude-mice	0.5 mg/kg; 30 days	Inhibition of pancreatic cancer growth	↑c-caspase 3 ↓PCNA, p-EGFR, p-AKT, Bcl-2	
Plant	Diosgenin	*Solanum, Dioscorea, Costus* species	Patu8988, PANC-1	50, 75 μM; 48 h	Induction of apoptosis	↑PTEN ↓EZH2, vimentin	[34]
			PANC-1 bearing mice	20 mg/kg; 4 weeks	Inhibition of tumor growth	↑PTEN ↓EZH2, vimentin	
Plant	Echinacoside	Stems of *Cistanchessalsa*	SW1990	20, 50, 100 µM; 5 days	Induction of apoptosis	↑ROS, Bax, p38 ↓MMP, JNK, ERK1/2	[35]
Plant	Elemene	*Zingiberaceae*	BxPC-3, PANC-1	15, 30, 60 μg/mL; 12 h	Inhibition of cell proliferation, Induction of cell cycle arrest		[36]
			BxPC-3 bearing BALB/c mice	20, 40, 60 mg/kg; 18 days	Inhibition of cell proliferation, Induction of cell cycle arrest	↑p53 ↓Bcl-2	
Plant	Grape seed proanthocyanidins (GSPs)	*Vitis vinifera*	PANC-1	20 µg/mL; 3, 12, 24 h	Induction of apoptosis	↓miRNA-SS3, SS12, SS24	[37]
						↓CDK6, EGFR, MSH6, DNMT1	
Plant	Hydroxychavicol	*Piper betle*	MIA PaCa-2	100 µM; 48 h	Induction of apoptosis	↑c-caspase -8, -9, c-Bid	[38]
			MIA PaCa-2, PANC-1	50, 100 µM; 48 h		↑c-caspase-3, -8, -9, c-Bid, c-PARP, Bax ↓Bcl-2, survivin	
Plant	Hyperoside		MIA PaCa-2	50 μM; 48 h	Induction of caspase-dependent apoptosis	↑c-caspase-3	[39]
	Hypoxoside		INS-1	25 μM; 48 h		↑c-caspase-3	
Plant	Icariin	*Herba Epimedii*	PANC-2	100, 150, 200 μM; 48 h	Induction of apoptosis		[40]
			PANC-2 bearing C57BL/6 mice	120 mg/kg; 11 days	Inhibition of pancreatic tumor progression		
Plant	Methyl4-(2-isothiocyanatoethyl)benzoate	Cruciferous vegetables	PANC-1	10 µM; 72 h	Induction of apoptosis	↑ROS ↓GSH	[41]
	N-Ethyl-4-(2-isothiocyanatoethyl)benzamide						
Plant	Mastic gum resin	*Pistacia atlantica*	PANC-1	20, 40, 60, 80, 100 µg/mL; 72 h	Induction of cytotoxicity		[42]
Plant	Monogalactosyl diacylglycerol	Spinach	MIA PaCa-2	25, 50, 75 μM; 24 h	Induction of apoptosis	↑cytochrome c, c-PARP, Bax, c-caspase-3 ↓Bcl-2	[43]
			MIA PaCa-2 bearing BALB/cAJcl-nu/nu mice	2 mg; 23 days	Inhibition of tumor growth		
Plant	Piperlongumine	*Piper longum*	BxPC-3, PANC-1, AsPC-1	5, 10, 20, 30, 40 μmol/L; 24, 48, 72 h	Induction of apoptosis Enhancement of gemcitabine-induced apoptosis	↑procaspase-3, c-PARP ↓Bcl-2, Bcl-xL, survivin, XIAP	[44]
			BxPC-3 bearing BALB/c mice	10 mg/kg; 21 days	Inhibition of tumor growth Enhancement of gemcitabine-induced apoptosis		
Plant	Piperlongumine	*Piper longum*	PANC-1	5, 10, 15 μmol/mL; 24, 48 h	Induction of apoptosis, Inhibition of cell proliferation	↓miR-27a, miR-17/miR-20a	[45]
						↑c-PARP, ROS	
			L3.6pL bearing athymic nu/nu mice	30 mg/kg; 21 days	Inhibition of tumor growth	↓miR-27a, miR-17/miR-20a	
						↓Sp1, Sp3, Sp4	
Plant	RN1	Flower of *Panax notoginseng*	AsPC-1, BxPC-3	62.5, 125, 250, 500, 1000 μg/mL; 48 h	Inhibition of PDAC cell growth	↓Galectin-3, EGFR, ERK, Runx1	[46]
			BxPC-3 bearing BALB/c nude mice	0.5, 20 mg/kg; 46 days		↓Galectin-3, Ki-67, EGFR, ERK, Runx1	
Plant	Rottlerin	*Mallotus phillippinensis*	Patu8988	4 μM; 48 h	Induction of apoptosis	↑cytochrome c ↓Skp2	[47]
			PANC-1	3 μM; 48 h			
Plant	Sugiol		MIA PaCa-2	7.5, 15, 30 µM; 48 h	Induction of apoptosis and cell cycle arrest Increase of ROS production	↑Bax ↓Bcl-2, MMP	[48]
Plant	Withaferin A	*Withania somnifora*	AsPC-1	1 μM; 48 h	Induction of apoptosis		[49]
Plant	Withaferin A	*Withania somnifera*	PANC-1, MIA PaCa-2	0.5, 1, 2.5, 5 μM; 24 h	Induction of apoptosis	↑c-caspase-3, -8, -9, c-PARP1	[50]
			PANC-1 bearing BALB/c mice	4 mg/kg; 24 days	Enhancement of the therapeutic efficacy of ER stress aggravators		
Plant	Bitter apricot ethanolic extract	*Prunus armeniaca* L.	PANC-1	704 μg/mL; 72 h	Induction of apoptosis	↑Bax, caspase-3 ↓Bcl-2	[51]
Plant	*Clinacanthus nutans* non-polar stem extracts (SN) and gemcitabine combination	*Clinacanthus nutans*	BxPC-3, SW1990	5 μg/mL (and/or 5 μg/mL of gemcitabine); 48 h	Induction of apoptosis Enhancement of gemcitabine-induced apoptosis	↑Bax ↓Bcl-2, cIAP-2, XIAP	[52]
Plant	Coix seed emulsion	*Coix lacryma-jobi*	BxPC-3	1.50–10 mg/mL; 48 h	Induction of apoptosis	↑caspase-3, c-PARP, Bax ↓Bcl-2, survivin, COX-2	[53]
			PANC-1	1.75–10 mg/mL; 48 h			
			AsPC-1	1.80–10 mg/mL; 48 h			
			BxPC-3 bearing nude BALB/c mice	12.5 mL/kg; 24 days		↓p65, Ki-67	
Plant	Cordifoliketones A	*Codonopsis cordifolioidea*	AsPC-1, BxPC-3, PANC-1	2, 4, 6 µg/mL; 24, 48 h	Induction of apoptosis	↑Bax, Bad, caspase-3, -8, -9 ↓Bcl-2, Bcl-xL	[54]
			AsPC-1, BxPC-3, PANC-1 bearing BALB/c nude mice	20, 80, 120, 240 M/kg; 27 days			
Plant	*Eucalyptus microcorys* aqueous extract (F1)	Leaf of *Eucalyptus microcorys*	MIA PaCa-2	100, 150 μg/mL; 48 h	Induction of cell cycle arrest and apoptosis	↑Bak, Bax, c-PARP, c- caspase-3 ↓Bcl-2, procaspase-3	[55]
Plant	*Eucalyptus microcorys* leaf aqueous extract	Leaf of *Eucalyptus microcorys*	MIA PaCa-2, BxPC-3, CFPAC-1	50, 100 μg/mL; 24 h	Induction of apoptosis		[56]
	*Eucalyptus microcorys* leaf ethanolic extract						
	*Eucalyptus microcorys* fruit aqueous extract	Fruit of *Eucalyptus microcorys*					
	*Eucalyptus saligna* ethanolic extract	*Eucalyptus saligna*					
Plant	Herbal mixture ethanol extract (H3)	*Meliae Fructus,* bark of *Cinnamomum cassia, Sparganium rhizome*	PANC-1	0.05 mg/mL; 72 h	Induction of apoptosis Induction of cell cycle arrest	↑cytochrome c	[57]
						↓COX-2, CXCR4, JAK2, XIAP	
			PANC-1 bearing BALB/c nude mice	200 mg/kg; 31 days	Inhibition of tumor growth	↑cytochrome c ↓COX-2	
Plant	*Inula helenium* L. ethyl acetate extract (EEIHL)	*Inula helenium* L.	CFPAC-1	2, 4, 6 µg/mL; 24 h	Inhibition of proliferation	↑p-AKT, p-STAT3	[58]
					Inhibition of cell migration	↑E-cadherin, c-PARP ↓Snail, XIAP,	
					Mitochondrial-dependent apoptosis	↑Bim ↓Bcl-2, Mcl-1	
Plant	*Moringa* aqueous leaf extract	*Moringa oleifera (Moringa)*	PANC-1	1.8 mg/mL; 24 h	Induction of apoptosis	↓Bcl-2, COX-2	[59]
			PANC-1 bearing CD-1 mice	0.5, 1.0, 1.5 mg/g; 6 weeks	Inhibition of tumor growth		
Plant	*Paeonia suffruticosa* aqueous extracts (PS)	*Paeonia suffruticosa*	PANC-1, AsPC-1, BxPC-3	750 µg/mL; 72 h	Induction of autophagy	↑caspase-3, -8, -9, c-caspase-3, DAPK3	[60]
			AsPC-1 bearing mice	0.9, 1.8 g/kg; 21 days	Inhibition of cell cycle progression and cell migration	↓Cyclin, CDK	
Plant	*Pterospermum acerifolium* ethanolic bark extract (PaEBE)	Bark of *Pterospermum acerifolium*	PANC-1	50, 75 µg/mL; 24 h	Induction of apoptosis	↑ROS	[61]
					Induction of mitochondrial-mediated cell death	↓MMP	
				50 µg/mL; 24 h	Induction of cell cycle arrest		
Plant	*Salvia chinensis* polyphenol-rich extract	*Salvia chinensis*	MIA PaCa-2	20, 40, 60, 80 µg/mL; 48 h	Induction of apoptosis	↑cytochrome c ↓MMP	[62]
Plant	*Sedum sarmentosum Bunge* extract (SSBE)	*Sedum sarmentosum Bunge*	PANC-1	100 µg/mL; 24 h	Induction of apoptosis	↑Bax, Bad, caspase-3, -8, p53	[63]
						↓Bcl-2, c-Myc, survivin	
			PANC-1 bearing BALB/c mice	10, 100 mg/kg; 30 days	Inhibition of tumor growth		
Plant	Total flavonoid aglycones extract	*Radix Scutellariae*	BxPC-3	3.2, 6.4, 12.8 μg/mL; 24 h	Induction of apoptosis	↑c-caspase-3, -8, c-PARP, c-Bid	[65]
			BxPC-3-bearing BALB/c nu/nu mice	50, 100, 150 mg/kg; 56 days	Induction of apoptosis and autophagy	↑c-caspase-3, c-PARP, LC3-II ↓p62	
Plant	F35 (alloalantolactone, alantolacton, isoalantolactone [1:5:4])	*Inula helenium* L.	PANC-1, SW1990	8 µg/mL; 48 h	Inhibition of proliferation		[66]
				6 µg/mL; 24 h	Induction of mitochondrion-related apoptosis	↑Bak ↓Bcl-2, Mcl-1, XIAP	
				2, 4 µg/mL; 24 h	Inhibition of colony-formation and migration		

GSH, Glutathione; c-caspase, cleaved caspase; Bax, Bcl-2-associated X protein; Bcl-2, B-cell lymphoma 2; PARP, Poly Adenosine diphosphate Ribose Polymerase; p-ERK, phospho-Extracellular-related kinase; p-c-JUN, phospho-c-Jun; pS6, phospho-S6; p-Bad, phosphor-Bad; COX-2, Cyclooxygenase-2; cIAP-2, Cellular inhibitor of apoptosis 2; XIAP, X-Linked Inhibitor of Apoptosis; DCLK-1, Doublecortin-like kinase 1; Shh, Sonic hedgehog signaling molecule; PCNA, Proliferating Cell Nuclear Antigen; EGFR, Epidermal Growth Factor Receptor; AKT, Protein kinase B(PKB); EZH2, Enhancer of Zeste Homolog 2; PTEN, Phosphatase and tensin homolog; ROS, Reactive Oxygen Species; MMP (ΔΨm), Mitochondrial membrane potential; E-cadherin, Epithelial cadherin; Mcl-1, Myeloid cell leukemia 1; CDK6, Cell division protein kinase 6; MSH6, MutS Homolog 6; DNMT1, DNA Methyltransferase 1; CXCR4, C-X-C Motif Chemokine Receptor 4; JAK2, Janus Kinase 2; c-Bid, cleaved-Bcl-2 homology 3 interacting domain death agonist; DAPK3, Death-Associated Protein Kinase 3, ↑—up-regulation; ↓—down-regulation.

In in vitro experiments, fifteen substances, 8-Chrysoeriol, elemene, mastic gum resin, RN1, bitter apricot ethanolic extract (BAEE), coix seed emulsion, *Eucalyptus microcorys* aqueous extract, *Eucalyptus microcorys* leaf ethanolic extract, *Eucalyptus microcorys* fruit aqueous extract, *Eucalyptus saligna* ethanolic extract, *Moringa* aqueous leaf extract, *Paeonia suffruticosa* aqueous extracts (PS), *Salvia chinensis* polyphenol-rich extract, *Pterospermum acerifolium* ethanolic bark extract (PaEBE), and *Sedum sarmentosum Bunge,* extract were conducted at a high concentration of 60 μg/mL or higher [31,36,42,46,53,56,59,60,61,62,63]. In such cases, when applied to humans, the amount of medicine could be far abundant, which requires attention. 

Besides inducing apoptosis, most of the substances had an inhibitory effect on tumor growth such as suppressing cell proliferation or inducing cell cycle arrest. Some substances were also effective in preventing metastasis, angiogenesis, and chemoresistance. Moreover, several natural products potentiated the anticancer activity of gemcitabine with a synergistic effect.

## 3. Metastasis Inhibiting Natural Products

To acquire metastatic features, cancer cells change their characteristics, leading to epithelial mesenchymal transition (EMT). EMT makes cancer cells to be founded in other tissues. Metastasis can be responsible for cancer-related death [67]. Thus, it is important to regulate this process, and we focused on both natural products and their compounds that contribute to anti-metastasis (Table 4).

Cheng et al. reported that *Poria cocos*-derived compound polyporenic acid and its extract have inhibitory effect of metastasis in PANC-1 cells [68]. A decrease in cell division cycle protein 20 homolog (CDC20) that has been unveiled to associate with invasion was observed by both treatments.

Zhang et al. reported that a novel natural compound terphenyllin has an anti-metastatic effect in both in vitro and in vivo [69]. A transwell assay and PANC-1 orthotopic model were used to measure the anti-cancer efficacies of terphenyllin. As a result, it was observed that treatment of terphenyllin reduced invasion as well as migration in HPAC and PANC-1. In vivo and histological data also showed the reduction of metastasis.

Novel compound cordifoliketones A, isolated from roots of *Codonopsis cordifolioidea*, showed an anti-cancer effect via the regulation of migration and invasion as well as apoptosis [54]. In the results of the invasion and migration assay, the reduction of invasion and migration in pancreatic ductal adenocarcinoma cells by cordifoliketones A was observed.

The expressions of C-X-C chemokine receptor type 4 (CXCR4) and cyclooxygenase-2 (COX-2) are increased after radiation therapy and facilitate metastasis of pancreatic cancer cells [70]. Aravindan et al. demonstrated that *Hormophysa triquertra* polyphenol (HT-EA) treatment represses irradiation-induced translation of CXCR4, COX-2, β-catenin, and matrix metalloproteinase-9 (MMP-9), Ki-67. Especially, repressions of CXCR4 and COX-2 lead to down-regulations of cancer cell migration and invasion. These results suggest that HT-EA would alleviate the dissemination of pancreatic cancer cells resistant to radiation-therapy.

Radiation has been commonly used for cancer therapy in decades. Hagoel et al. reported that combined with radiation and *moringa* aqueous leaf extract showed synergistic inhibitory activity in metastasis of PANC-1 cells [59]. As for migration and invasion, *moringa* treatment inhibited by 61.6% and 63.7% compared to control, respectively. Moreover, reductions in migration (56.4%) and invasion (39.8%) were observed by *moringa* combined with 4 Gy in comparison to control.

Dephosphorylation of cofilin by slingshot homologs (SSH) associates with actin depolymerization [71]. This change in actin dynamics can be responsible for invadopodia, protrusions observed invasive cancer cell. Lee et al. reported that a novel compound, sennoside A, contributes to a reduction in metastasis by acting as a slingshot inhibitor. After treatment, up-regulation of p-cofilin was confirmed in MIA PaCa-2 and PANC-1 cells. Furthermore, an in vivo study trans-planting PANC-1 cell into the spleens showed that sennoside A induced a prominent reduction of liver metastasis compared to control.

Pei et al. investigated whether a natural product toosendanin has anti-cancer activity on pancreatic cancer and reported that inhibition of migration and invasion was observed by this natural product both in vitro and in vivo [72]. The levels of E-cadherin, known as epithelial marker, increased, whereas those of mesenchymal markers, including vimentin, Snail, and ZEB, decreased. In addition, it was found that toosendanin attenuated the phosphorylation of AKT and mTOR as well as PRAS40 and p70S6K.

**Figure 2:**
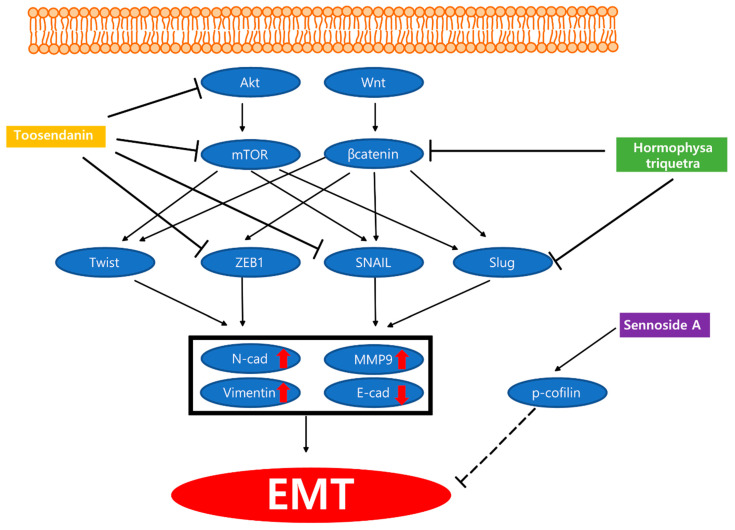
Schematic diagram of anti-metastasis mechanisms of various natural products against pancreatic cancer.

To sum up, eight substances derived from plants and fungi showed anti-metastatic activities against pancreatic cancer. The anti-metastatic mechanisms of natural products were illustrated in Figure 2. When compared to studies on apoptotic effect of natural substances, studies on metastasis are scarce. Among them, Poria co-cos showed that its extract and their compound polyporenic acid exert anti-metastatic potency. Moreover, CDC20, commonly known as cell division cycle associated factor, has been shown to participate in invasion. However, data concerning whether the empirical doses are toxic against normal cell lines remain necessary. The study of *Hormophysa triquetra* polyphenol elucidated that the polyphenol inhibited cell migration of pancreatic cancer cells In vitro. However, when 100 µg/mL of polyphenol was applied on PANC-1, PANC-3.27, BxPC-3, and MIA PaCa-2, a somewhat high concentration of over 60 µg/mL was observed. Treatment of *moringa* leaf extract with PANC-1 inhibited metastasis, but a synergistic effect was observed when combined with radiation. This suggests that natural products and their compounds combined with radiation could be effectively used for cancer therapy. Reflecting a partial interaction, the in vitro study has a limitation in that it does not represent whole sophisticated interactions. Thus, subsequent studies comprising an in vivo model appear to be needed. Of the studies mentioned above table, Pei et al. presented evidence supporting the anti-cancer activity of toosendanin from a molecular perspective both in vitro and in vivo. Notably, this study showed hallmarks of EMT, including Snail, vimentin, and E-cadherin.

## 4. Angiogenesis Inhibiting Natural Products

Angiogenesis is the formation of new blood vessels, and the tumor is provided with oxygen and nutrition by newly formed vessels. This is attributed to the growth of cancer cells and metastasis to other organs [73]. Thus, it is essential to down-regulate angiogenesis in cancer therapy. Various natural products have been demonstrated to regulate angiogenesis (Table 5).

Danggui-Sayuk-Ga-Osuyu-Saenggang-Tang (DSGOST) is a mixture of herbal extract used in Traditional Korean medicine [73]. Human umbilical vascular endothelial cells and human dermal microvascular endothelial cells were treated with DSGOST 100 µg/mL for 72 h with/without 50 ng/mL of vascular endothelial growth factor (VEGF). The treatment inhibited VEGF-dependent tube formation in both cases. In PANC-28 xenograft BALB/c nude mice model, injection of DSGOST at a dose of 100 μg with 100 ng/μL of VEGF down-regulated newly formed vessel number in the tumor, as measured by vascular permeability assays. For bioluminescence imaging analyses, DSGOST was orally administered at a dose of 20 mg/kg and repressed tumor growth. These results indicate that DSGOST mitigates vascular leakages caused by VEGF and thereby inhibits tumor growth.

SH003 is a mixture that contains *Astragalus membranaceus*, *Angelica gigas*, and *Trichosanthes Kirilowii* Maximowicz [74]. Choi et al. investigated whether the SH003 inhibits VEGF-induced tumor angiogenesis. Human umbilical vein endothelial cells were treated with SH003 at doses of 10, 20, and 50 µg/mL for 24 h, and VEGF was prevented from binding to VEGFR2. As a result, cell migration, invasion, and tube formation were suppressed through this mechanism. As assessed by vascular leakage assays in vivo PANC-28 bearing nude mice model, VEGF-induced vessel permeability was inhibited after an injection of SH003 at 20 μg. When SH003 was orally administered at a dose of 2 mg/kg, a reduction of tumor growth was detected in bioluminescence imaging analyses, while the bodyweight of the mice was not affected.

There were two types of research associated with anti-angiogenesis effect. DSGOST and SH003 were mixtures of various kinds of natural products and all of their contents were derived from plants [73,74]. Both materials were investigated by the same researchers who mainly investigated VEGF-related mechanisms. DSGOST and SH003 were treated to HUVECs in vitro and to PANC-28 bearing BALB/c nude mice in vivo. Both were demonstrated to down-regulate the metastasis by inhibition of angiogenesis without any effect on pancreatic tumor cell viability. In the case of DSGOST, inhibition of angiogenesis was observed at an extremely high concentration 100 µg/mL, higher than 60 µg/mL. Moreover, studies to investigate cytotoxicity to normal cells are required for DSGOST and SH003.

## 5. Resistance Inhibiting Natural Products

**Figure 3:**
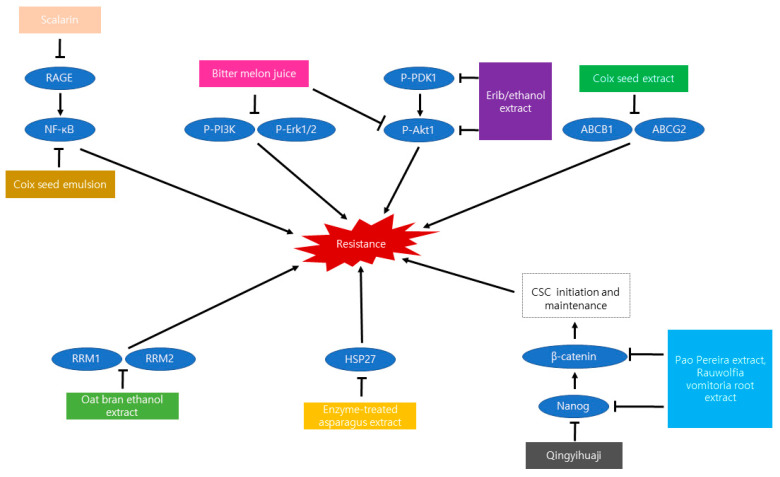
Schematic diagram of anti-resistance mechanisms of various natural products against pancreatic cancer.

Although various therapies have been developed against cancer, including chemotherapy, radiation therapy, and so forth, pancreatic cancer is still deadly and represents 10% of the five-year survival rate [75]. Resistance is known to occur in gemcitabine treatment, which is the most common anti-cancer drug [76,77]. The drug resistance makes gemcitabine ineffective, and it consequently leads to difficulties in the successful treatment of cancer. For this reason, only 12% of patients treated with gemcitabine showed anti-cancer efficacy [78]. Therefore, new substances which enhance the efficacy of the anti-cancer drug and suppress drug resistance are needed [79]. There were 10 natural products demonstrated to regulate factors inducing resistance and promote the efficacy of anti-cancer therapy (Table 6). The anti-resistance mechanisms of natural products were illustrated in Figure 3.

Terpinen-4-ol is a kind of monoterpene derived from mainly tee-tree oil [80]. Shapira et al. investigated the anti-cancer effect of terpinen-4-ol on pancreatic cancer in vitro and in vivo. A synergistic tumor growth inhibition effect was observed when the compound was combined with oxaliplatin, fluorouracil, or gemcitabine, which are the general anticancer medicines. Especially, when gemcitabine was combined with terpinen-4-oil at a concentration of 0.01%, the tumor growth inhibition effect was increased to 60–85%. In vivo, this substance reduced the tumor volume and weight in the colorectal DLD1 cancer cell xenograft model. Moreover, it was demonstrated that terpinen-4-ol inhibited colorectal, gastric, even prostate cancer growth as well as the growth of pancreatic cancer.

Scalarin, derived from *Euryspongia* cf. *rosea,* showed a reduction of receptor for advanced glycation end products (RAGE) [81]. RAGE level was down-regulated when PANC-1 and MIA PaCa-2 were exposed to 10 µg/mL of scalarin for 24 h. This result suggests that scalarin diminishes the drug resistance and proliferation of pancreatic cancer cells. However, RAGE-associated factors, such as Bcl-xL, p-ERK 1/2, p-NFκB, and p-STAT3, were not modified by scalarin, so researchers suggested that it may be because of other mechanisms associated with RAGE.

Somasagara et al. demonstrated that an increase of phosphorylated AKT and ERK1 is associated with gemcitabine resistance [82]. Bitter melon juice inhibited the phosphorylation of AKT and ERK1/2. When gemcitabine-resistant AsPC-1 cells were treated with bitter melon juice, cell viability was diminished through targeting AKT mediated pathway.

Activation of NF-κB induced by gemcitabine was abolished by pretreatment of coix seed emulsion (CSE) [53]. CSE pretreatment, as agents which down-regulate NF-κB, reduced resistance to gemcitabine and increased sensitivity to gemcitabine in pancreatic cancer cells. Such pretreatment also activated proteins which induce apoptosis, such as caspase-3, c-PARP, and Bax, and reduced counteracting factors, such as Bcl-2, survivin, and COX-2. Consistent with in vitro study, CSE administration combined with gemcitabine treatment indicated a 68% decrease of tumor weight on BxPC-3 xenograft models at a dose of 12.5 mg/kg, which is superior to each treatment alone.

Gemcitabine-induced ABC transporters were previously reported to cause drug resistance [76]. Qian et al. demonstrated that coix seed extract downregulated the expression of ABCB1 and ABCG2 and induced sensitivity to gemcitabine. The coix seed extract treatment elevated gemcitabine accumulation in BxPC-3 cells, while down-regulating drug efflux and elimination. In BxPC-3 and PANC-1 at a dose of coix seed extract 10 mg/mL for 24 and 48 h combined with gemcitabine 3 µg/mL, the level of IC50 of gemcitabine was significantly decreased. This result suggests that the compound induces a synergistic anti-cancer effect with gemcitabine.

Enzyme-treated asparagus extract (ETAS) is known to induce heat-shock protein (HSP) 70 [77]. Shimada et al. investigated the regulation effect of ETAS on HSP27, which was previously demonstrated to causes gemcitabine resistance. When ETAS was treated to gemcitabine-resistant KLM1-R cell line at 2 mg/mL for 120 h, the expression of HSP27 and p-HSP27 (serine 78) were mitigated. This result suggests that ETAS would show a synergistic effect with gemcitabine and would be used to induce the sensitivity of gemcitabine on drug-resistant cancer cells.

Based on previous studies of the effectiveness of Eriocalyxin B (EriB) in treating pancreatic cancer, Li et al. studied whether EriB could assist the function of gemcitabine against SW1990 cells [79]. EriB, the ethanol extract of *Isodon eriocalyx*, stimulated gemcitabine-induced apoptosis and anti-proliferative effect. Among the mixtures of gemcitabine and EriB in various concentrations, 25 µM gemcitabine/1.25 µM EriB combination exhibited a remarkably synergistic effect on SW1990 cells. Especially when 2.5 µM of EriB was treated alone, phosphorylation of AKT was diminished, whereas AKT and PDK1 were both inhibited from phosphorylation when EriB was combined with 25 µM of gemcitabine.

Oat bran ethanol extract (OBE) was elucidated to overcome drug resistance by reduction of RRM1 and RRM2 levels, which was expressed higher in MIA PaCa-2 cells treated with gemcitabine than those were not [78]. The viability and proliferation of gemcitabine-resistant pancreatic cancer cells were selectively disrupted, as compared with normal pancreatic cells. Additionally, OBE induced the apoptosis of pancreatic cancer cells by the activation of AMPK and inactivation of JNK. Consistent with this, OBE increased the cell accumulation in the G0/G1 phase and death of pancreatic cancer cells in flow cytometry analysis and a TUNEL assay of MIA PaCa-2 and PANC-1 cells.

Another study of Pao Pereira extract derived from *Geissospermum vellosii*, was conducted to investigate the inhibitory effect on tumor spheroid formation by Dong et al. [83]. The anti-resistance effect of the Pao Pereira extract was proved by examining pancreatic cancer stem-like cells (CSCs) surface markers and tumor spheroid assay. As a result, surface markers such as CD44+ CD24+ EpCam+ were diminished, and IC50 of Pao was 27 µg/mL in spheroids inhibition. At a higher concentration of 100 μg/mL, it completely inhibited spheroid formation on PANC-1 and MIA PaCa-2. Pao reduced β-catenin and Nanog by down-regulation of BCL2L2, COX-2, and several kinds of mRNA levels related to CSCs, such as Dppa4, Esrrb, and Tcl1. In vivo, the expression of CSCs and tumorigenicity were reduced in the PANC-1 xenograft nude mice model. However, the CSCs inhibitory mechanism of Pao was also not clearly identified in the present study.

Qingyihuaji is an extract of five herbs; *Scutellariae barbatae*, *Hedyotdis*, *Arisaematis erubescentis, Gynostemmatis pentaphylli*, Amomi Rotundus [84]. Chen et al. reported that Qingyihuaji elevated the expression of lncRNA AB209630 while inhibited that of miR-373, EphB2, Nanog. Gemcitabine-resistant CFPAC-1 treated with Qingyihuaji combined 30 ng/L of gemcitabine showed synergistic effects on cell proliferation and migration. In vivo, the combination of Qingyihuaji and gemcitabine showed a greater antitumor effect than separately treating them.

CSCs are related to cause drug resistance and metastasis in pancreatic cancer [85]. Dong et al. reported that *Rauwolfia vomitoria* root extract treatment reduced β-catenin and Nanog which initiate and maintain CSCs. The reduction of β-catenin and Nanog indicates that the compound inhibits tumor spheroid formation of CSCs. The extract exerted complete inhibition of the tumor spheroid in PANC-1 at a dose of 200 µg/mL, and in MIA PaCa-2 at a higher concentration of 100 µg/mL. At lower than each concentration, a reduction of tumor spheroid was shown in both PANC-1 and MIA PaCa-2. It also was demonstrated to suppress tumor formation in a NCr-nu/nu mice model at a dose of 20 mg/kg.

Scalarin was originated from a marine sponge whereas the remaining ten were from the plant [81]. Terpinen-4-ol was the only one effective at resistance to anti-EGFR therapy [80]. Coix seed emulsion and coix seed extract were extracted from the same natural product [53,76]. Moreover, there was no statement about sources of ETAS [77]. When OBE was treated on hTERT-immortalized human pancreatic epithelial nestin-expressing (HPNE) cells obtained from a normal pancreatic duct, cytotoxicity was not found at less than 40 µg/mL [78]. Both *Rauwolfia vomitoria* root extract and Pao Pereira extract had less impact on MRC-5 epithelial cells that are not cancer cells than pancreatic cancer cells at concentrations of 567 µg/mL and 547 μg/mL [83,85]. Terpinen-4-ol, scalarin, bitter melon juice, coix seed emulsion, coix seed extract, ETAS, EriB/ethanol extract, and Qingyihuaji were not tested for confirmation of cytotoxicity in normal cells [53,76,77,79,80,81,82,84]. There was a lack of in vivo tests regarding scalarin, bitter melon juice, OBE, ETAS, and EriB/ethanol extract [77,78,79,81,82]. Additional studies about these need to be carried out in order to be used for an adjuvant to clinical chemotherapy.

## 6. Clinical Trials

There were seven clinical trials of natural products against pancreatic cancer from 2009 to 2020, and five types of natural products were tested (Table 7). Phase 1–3 pancreatic cancer clinical trials were targeted, and a majority of the clinical trials of phase 2 were conducted. Most of them were completed, while one was yet recruiting participants.

In a study, 15 patients with gemcitabine-refractory advanced pancreatic cancer were the subjects of UMIN000005787, a clinical trial that took place in Japan [86]. GBS-01, extracted from the fruit of *Arctium lappa* L., was administered orally once a day until the disease progresses or unacceptable toxicity occurs. None of the patients treated with GBS-01 showed any signs of dose-limiting toxicities (DLTs). The main adverse effects were the increase of γ-glutamyl transpeptidase and hyperglycemia. The trial was a non-randomized, unblinded, and uncontrolled study, executed from 16 June 2011 to 5 May 2014. According to the clinical trial results, the recommended dose of GBS-01 was 12.0 g q.d, and the clinical safety of GBS-01 monotherapy was confirmed in pancreatic cancer patients who were difficult to treat with gemcitabine therapy [87]. The co-treatment of gemcitabine and GBS-01 can also be a promising therapy for pancreatic cancer patients, as gemcitabine has anti-tumor effects on cancer cells under oxygen and glucose-rich conditions, while GBS-01 has the ability to remove the resistance of cancer cells.

Non-randomized, open-label study NCT00192842 expected that curcumin, extracted from *Curcuma longa Linn*, could aid the efficacy of gemcitabine [88]. The patients were administered gemcitabine once a week and had 8 g of curcumin orally every day. The study started in July 2004 and was primarily completed in November 2007. The result of this trial was not posted.

The phase 2 clinical trial NCT00094445 started on November 2004 and results were updated on 28 August 2020 [89]. A total of 50 patients suffering from pancreatic cancer were administered 8 g of oral curcumin every day for eight weeks. In total, 44 patients completed the study. About 20% of the patients had severe adverse events including cardiac disorders (chest pain, multiple pulmonary emboli, atrial fibrillation etc.), gastrointestinal disorders (GI hemorrhage, abdomen pain etc.), dehydration, or pain. About 13% of the patients had no serious adverse events like gastrointestinal disorders (vomiting, nausea) or edema.

The aim of the phase 2 clinical trial NCT00837239 was to compare the effect of two types of treatment against pancreatic cancer: the combination of HuaChanSu plus gemcitabine, and gemcitabine plus placebo [90]. Both groups were administered 1000 mg/m^2^ gemcitabine once a week and then skipping a week, for a cycle of 28 days. One group was additionally treated with HuaChanSu, 20 mL/m2 for a total 500 mL, given 2 h infusion, 5 days a week (3 weeks), and skipping a week. The other group was the placebo group, additionally treated with saline. It was a randomized, placebo-controlled, and blinded study, started in June 2007 and primarily completed in July 2012. According to the results of randomized clinical trials, locally advanced pancreatic or metastatic pancreatic cancer patients’ outcomes were not improved when treated with the combination of huachansu and gemcitabine [91]. So, the co-treatment of hwachansu and gemcitabine against advanced pancreatic cancer is not recommended. Kanglaite, an oil extract from *Coicis Semen*, was used in phase 2 NCT00733850 clinical trial [92]. It was targeted to 85 adults suffering from pancreatic cancer. The experimental group was administered kanglaite injection plus gemcitabine, and the compared group was administered only gemcitabine. This was a randomized, open-label, interventional study, that occurred from August 2008 to June 2014. The clinical trial report was sent to the FDA, and the phase 3 clinical trial is expected. According to the results, the combination of 30 g kanglaite injection per day and a standard therapy of gemcitabine has been demonstrated to encourage clinical evidence of proliferation effects and a well-controlled safety profile [93].

The phase 3 clinical trial ISRCTN70760582 targeted 434 adults suffering from four pancreatic cancers [94]. Mistletoe therapy was predicted to enhance the survival rate and improve the life quality of the patients. Iscador Qu Spezial (IQuS), subcutaneous injection of an extract of *Viscum album* L., was given to the patients three times a week for 12 months, and all patients were followed up for 12 months. This trial started on 1 January 2009 and ended on 31 December 2014. According to the result, mistletoe is an effective supplementary treatment for locally advanced or metastatic pancreatic cancer in that mistletoe treatment noticeably improved the quality of life in patients suffering from the disease [95].

Iscador Qu, extracted from *Viscum album* L., is planned to be used to pancreatic cancer patients in phase 3 clinical trial NCT02948309 [96]. This study is present recruiting patients and is estimated to be completed in June 2021. Half of the patients will be administered Iscador Qu by subcutaneous injections and the other half will be the placebo group. The result of the trial was not posted.

There are seven clinical trials for treating pancreatic cancer using natural products. Those conducted in the US were five: two of them published in the last 10 years, three of them in the last five years. One was conducted in the UK, 11 years ago, the other one in Japan, six years ago. Curcumin and Iscador Qu (Mistletoe extract) were tested twice each. One of the studies was phase 1, four were phase 2, and two were phase 3. Six out of seven trials were completed, and four of them posted results. There were evident gaps in the number of participants among the clinical trials. Four clinical trials had recruited more than 80 people, while two trials had about 15 participants. In three out of seven trials, Curcumin and GBS-01 were administered orally, and the rest were administered by subcutaneous injections.

**Table 7:** Clinical trials of natural products against pancreatic cancer.

Compound/Extract	Source	Phase	Patients	Status	Results	Registry Number	Reference
GBS-01	Fruit of *Arctium lappa*	Phase 1	Pancreatic Cancer (≥20 yrs) 15 participants	Completed	Safe up to 12 g per day Favorable response	UMIN000005787	[86]
Curcumin	*Curcuma longa Linn*	Phase 2	Pancreatic Cancer (≥18 yrs) 17 participants	Completed	Partial response 1 Stable disease 4 Tumor progression 6 Discontinued 5 Dose reduced 2	NCT00192842	[88]
Curcumin	*Curcuma longa Linn*	Phase 2	Pancreatic Cancer (≥18 yrs) 50 participants	Completed	The six-month survival rate was 15.9% (7/44)	NCT00094445	[89]
Huachansu/Bufo toad skin water extract	*Bufo gargarizans*	Phase 2	Pancreatic Cancer (≥18 yrs) 80 participants	Completed	Median overall survival Experimental: 160 days Control: 156 days Not statistically significant (*p* = 0.339)	NCT00837239	[90]
Kanglaite/oil extract	*Coicis Semen*	Phase 2	Pancreatic Cancer (≥18 yrs) 85 participants	Completed	Progression-free survival Experimental: 112 days Control: 58 days	NCT00733850	[92]
Iscador Qu Spzial	*Viscum album*	Phase 3	Pancreatic Cancer (≥18 yrs) 434 patients	Completed	Mistletoe treatment improve the global health status significantly	ISRCTN70760582	[94]
Iscador Qu/Mistletoe extract	*Viscum album* L.	Phase 3	Pancreatic Cancer (≥18 yrs) 290 participants	Recruiting	N/A	NCT02948309	[96]

N/A, not available.

## 7. Discussion

Despite continuous research on the treatment of pancreatic cancer, the proportion of patients overcoming pancreatic cancer has not improved noticeably. Therefore, recognizing a novel treatment is needed, and we found that the therapy of pancreatic cancer using the natural products has been meaningful. We analyzed original research in terms of apoptosis, anti-metastasis, anti-angiogenesis, and anti-resistance. Additionally, we reviewed the clinical trials practiced in various countries.

### 7.1. Anti-Cancer Mechanisms of Natural Products

In total, 68 drugs were reviewed for their anti-cancer effects against pancreatic cancer. The mechanisms of anticancer were divided into four categories, namely apoptosis, anti-metastasis, anti-angiogenesis, and anti-resistance, with a significant proportion of drugs related to apoptosis. MIA PaCa-2, PANC-1, AsPC-1, or BxPC-3 pancreatic cancer cell lines were usually used in in vitro studies, with several studies including in vivo experiments, usually using BALB/c mice as an animal model.

Apoptosis, known as programmed cell death, plays a significant role in health and disease [22]. Inappropriate apoptosis may occur in various diseases, including many types of cancer like pancreatic cancer. However, not all researches had in vivo studies, but 17 were effective in a mice model [24,27,33,34,36,40,43,44,45,46,50,53,54,57,59,60,63,65]. Fifteen substances were administrated at a high concentration of 60 μg/mL or higher, which demands attention when applied to humans [31,36,42,46,51,53,55,56,59,60,61,62,63]. Twenty substances conducted cytotoxicity experiments [24,26,29,30,36,38,41,42,43,44,46,49,50,51,53,55,62]. The rest of the studies require additional toxicity tests to normal pancreatic cancer cells. In the case of leiodermatolide, it showed low efficacy on cell survival [27]. Even though the administration of leiodermatolide exhibited potent cytotoxicity compared to control groups or gemcitabine groups, it was not completely preventable due to the possibility of toxicity. If administered less, it will be possible to increase the survival rate and get accurate results for efficacy. Notably, 8-Chrysoeriol as a natural dietary product potentially targeting BCL-2 could serve as a lead compound for SW1990 pancreatic cancer therapy [31]. In addition, echinacoside (ECH) induced apoptosis by elevating ROS and reducing MMP in SW1990 cells [35]. However, since SW1990 cell lines do not have the mutation of p53, which triggers the upregulation of Bax, additional investigation is needed to determine whether ECH is available for suppressing tumors.

Metastasis, the tendency of cancer cells to spread to different organs, is a sign of cancer that is distinguished from benign tumors [97]. Seven natural products that originated from plants and fungi were observed to have anti-metastasis-related mechanisms in pancreatic cancer. Six compounds had one or no mechanism, which encourages additional studies about specific mechanisms. Still, it is widely known that the downregulation of CDC20 plays an important role in the cell cycle. Several reports described inhibition of metastasis in both cell lines and in vivo [69,71,72]. However, the remaining studies require in vivo experiments. Moreover, *Poria cocos* extract and its compound polyporenic acid seem to require data indicating whether the empirical doses are noxious to normal cell lines. About half of the products were administered to the cancer cell line at an abnormally large amount, treated with a dose of 60 μg/mL or more [59,68].

Angiogenesis, which supports the growth of cancer cells and metastasis to other organs, should be down-regulated when treating cancer [73]. Two compounds included successful in vivo experiments, but there was no cytotoxicity test [73,74]. One out of two compounds was tested with a concentration of 100 µg/mL [73].

Since pancreatic cancer is highly resistant to chemotherapy and radiation therapy, anti-resistance is one of the decisive factors in cancer treatment [70]. Ten natural products that are related to anti-resistance were reviewed. Half of the studies not only included in vitro experiments, but also in vivo experiments [53,70,76,85]. Moreover 60% of the compounds were administered in high doses on cancer cell lines, which demands attention when applied to humans [53,70,76,77,83,85]. Notably, coix seed extract (10 mg/mL), coix seed emulsion (4.0 mg/mL), and enzyme-treated asparagus extract (2 mg/mL) were way beyond the standard, 60 μg/mL. Pao Pereira extract and *Rauwolfia vomitoria* root extract were assessed in highly reliable studies conducted in vitro, in vivo, and even with normal cytotoxicity tests.

### 7.2. Clinical Trials of Natural Products against Pancreatic Cancer

Although there is original research about 68 products in this review, seven clinical trials have been given a registry number and internationally recognized. Even if the substances used in clinical trials were not included in this review, they have been experimentally studied for their anti-cancer effects for a long time or have been used in clinical trials for cancers other than pancreatic cancer [98,99,100,101]. Therefore, among the studies in this review, particularly highly reliable substances are worth testing in clinical trials.

Since Pao Pereira extract has various anti-cancer effects, such as inducing apoptosis, increasing sensitivity to gemcitabine, and suppressing tumor growth in vivo, a clinical trial dealing with the administration of Pao Pereira extract plus gemcitabine is recommended. *Isodon eriocalyx* ethanol extract showed good effects in induction of apoptosis and anti-resistance, and also was effective in suppressing proliferation. Thus, clinical trials can be conducted if successful results are achieved in in vivo studies.

### 7.3. Multi-Functional Natural Products

Among the natural products, several products were multi-functional natural products, which have treated pancreatic cancer through two or more mechanisms. Emulsion and extract from *Coix lacryma-jobi* showed an outstanding effect in the treatment of pancreatic cancer in apoptosis and anti-resistance. In particular, the reliability of both studies was raised by including in vivo experiments as well as in vitro experiments. Although abnormally high doses were administered in vitro, emulsion or extract from *Coix lacryma-jobi* have been the subject of completed clinical trials as kanglaite and proven to be acceptable to humans. Natural products that treat pancreatic cancer through apoptosis and anti-metastasis are Cordifoliketones A and *Moringa* aqueous leaf extract. In anti-metastasis, both extracts require additional experiments due to the lack of in vivo experiments. *Moringa* aqueous leaf extract showed great anti-cancer effects, especially when combined with radiation therapy. In addition, many of the natural substances in anti-resistance had other effects, such as the induction of apoptosis or inhibition of tumor growth as well as the effect on resistance. Oat bran ethanol extract (OBE), *Isodon eriocalyx*, Coix seed emulsion, *Rauwolfia*, and Terpinen-4-ol were demonstrated to induce the apoptosis of pancreatic cancer cells. *Isodon eriocalyx* was especially also effective in suppressing proliferation. Furthermore, *Pao Pereira* extract was demonstrated to induce apoptosis in a previous study. According to the experimental study reviewed in this paper, *Pao Pereira* extract was proven to increase sensitivity to gemcitabine apart from the induction of apoptosis, suppressing tumor growth.

### 7.4. Anti-Inflammatory and Anti-Tumor Effects of Natural Products

The mechanisms of the reviewed natural products suggest that they are somewhat related to anti-inflammatory activity. Resveratrol acts to activate anti-inflammatory reactions through modulation of enzymes and pathways that produce mediators of inflammatory reactions, and also induces programmed cell death in activated immune cells. Resveratrol, which has no side effects even at high concentrations, has significant potential to be used as an alternative therapy against cancer and inflammatory diseases [14,15,102]. Curcumin is known to have a significant anti-inflammatory effect by blocking NF-κB signals at several stages, and shows great potential in the anti-tumor effect by inhibiting I-κB kinase [16,103]. It can be seen shown that anti-inflammatory mechanisms, including the NF-κB mechanism of natural products and natural products with anti-inflammatory effects, are strongly related.

### 7.5. miRNA Regulating Natural Products 

The manipulation of miRNAs is known to be useful in the treatment of diseases, especially cancer [16,37]. Therefore, in the present review, we noted the papers related to miRNAs, but this had drawbacks in that only two out of the 68 natural products considered involved mechanisms of miRNAs, which were concentrated on apoptosis [37,45]. Additionally, poorly regulated miRNAs have the potential to cause pancreatic cancer, the dysregulated miR-646 and MIIP expression being associated with the exacerbation of pancreatic cancer [104].

### 7.6. Strengths and Limitations

In this review article, various anti-cancer mechanisms of pancreatic cancer were systematically analyzed and the source, cell line, animal model, dose, duration, efficacy, and mechanism of the natural compound in each paper were arranged clearly. Notably, the tables were divided into seven sections based on the anti-cancer mechanism and the sources of natural products. Anti-tumor activity by modulating miRNAs was separated from other mechanisms. Most of the reviews in the last 10 years were based on immunotherapy. Yue et al. reported a similar review about treating pancreatic cancer with the combination of natural products and chemotherapeutic agents like gemcitabine [105]. They only reviewed the studies that had the anti-cancer mechanism of apoptosis, but we analyzed the studies of the four different anti-cancer mechanisms and the clinical trials additionally. In particular, we not only dealt with the study of synergetic effect with anti-cancer drugs, but also focused on the inherent anti-cancer efficacy of natural products. In addition, pancreatic cancer treatment using natural products has a great advantage of being effective in various mechanisms and producing synergistic effects. Fortunately, there were no papers on natural products that cause pancreatic cancer.

Limitations exist in this review, which does not include papers older than five years after publication, and only studies written in English have been reviewed. In addition, the research on compounds and extracts was mostly reviewed. Moreover, among the reviewed papers, in vitro experiments were performed in all the cited studies, but not all of the papers conducted in vivo experiments, so further investigation is suggested. The possibility that the concentrations of natural substances that were effective in in vitro experiments may not be effective in actual clinical practice cannot be excluded. Since natural products have multi-compound and multi-target characteristics, additional mechanistic studies are needed, and further studies on bioavailability and drug delivery systems are also needed.

### 7.7. Future Research Directions for Natural Product Treatment against Pancreatic Cancer

In future studies on pancreatic cancer treatment, studies on synergistic effects related to existing anti-cancer drugs or studies on natural products that suppress side effects of anti-cancer drugs are needed. Natural products that can treat various symptoms, such as loss of appetite and weight loss related to cachexia due to pancreatic cancer, should be studied. When the natural products of the studies and clinical trials were searched at nifds.go.kr, several drugs were found to have been used as traditional medicines in China, Japan, and Korea. These drugs suggest that modern cancer treatment ideas can be obtained from traditional medicine [34,44,45,57,58,60,66,68,73,74,86,88,89,94,96]. Additionally, since there have been many studies on the effects of natural products inhibiting resistance, this field is promising and noteworthy.

## 8. Methods

Original research regarding the effects of TKM or natural products on pancreatic cancer were collected from PubMed and Google scholar. When searching for relevant original research, we included “natural product”, “pancreatic cancer”, “apoptosis”, “metastasis”, “angiogenesis”, and “cancer drug resistance” as keywords. After the initial search, we only included studies between July 2015 and April 2020. We selected studies that fit into the following criteria: (1) research based on in vitro or in vivo to demonstrate anti-cancer effects of natural products; (2) research containing experiments using natural products derived from fungi, marine sponges, or plants; (3) statically significant researches whose *p* values were less than 0.05; (4) researches written in English.

Clinical trials about natural products against pancreatic cancer were collected from clinicaltrials.gov, isrctn.com, and umin.ac.jp/ctr/. Clinical trials conducted in other countries rather than the USA were additionally searched in PubMed. When searching for clinical trials, “pancreatic cancer” and “natural product” were used as keywords. We only included studies between February 2009 and April 2020. Subsequently, we collected clinical trials suitable to the following criterion: (1) clinical trials that include administration of natural-derived treatment.

## 9. Conclusions

In this study, 68 natural products that treated pancreatic cancer were classified and organized by their various anti-cancer mechanisms. The anti-cancer effects of natural products and regulations in related factors for each anti-cancer mechanism were easily summarized. Therefore, the recent research trend in pancreatic cancer treatment using natural products can be easily recognized. Natural products have high potential in treating pancreatic cancer by having a synergetic effect with conventional anti-cancer drugs and inherent anti-cancer efficacy themselves. Nevertheless, many cases are limited to in vitro studies, and for natural products to make a greater contribution to the treatment of pancreatic cancer, further in vivo studies and clinical trials are needed.

## Figures and Tables

**Table 1:** Apoptosis inducing fungi.

Classification	Compound/ Extract	Source	Cell Line/Animal Model	Dose; Duration	Efficacy	Mechanism	Reference
Fungus	*Agaricus blazei* Murrill water extract	*Agaricus blazei* Murrill	MIA PaCa-2, PCI-35, PK-8	0.005, 0.015, 0.045%(*w*/*v*); 48 h	Induction of apoptosis	↑c-caspase-3, -9, c-PARP	[23]
Fungus	Chaetospirolactone	*Chaetomium* sp. NF00754	HPDE6c-7, AsPC-1, PANC-1	100 nM; 18 h	Induction of apoptosis	↑c-caspase-3, -8, -9 ↓EZH2	[24]
			AsPC-1-bearing BALB/c mice	0.075 mg/kg; 28 days		↑c-caspase-3	
Fungus	Dicatenarin	*Penicillium pinophilum*	MIA PaCa-2	20 µg/mL; 48 h	Induction of apoptosis	↑cytochrome c, caspase-3	[25]
Fungus	Skyrin	*Penicillium* *pinophilum*	MIA PaCa-2	50 µg/mL; 48 h	Induction of apoptosis	↑cytochrome c, caspase-3	
Fungus	Xylarione A	*Xylaria psidii*	MIA PaCa-2	10, 30, 50 µm; 24 h	Induction of apoptosis	↓MMP	[26]
	(-) 5-methylmellein						

c-caspase, cleaved caspase; PARP, poly adenosine diphosphate ribose polymerase; MMP (ΔΨm), Mitochondrial membrane potential; ↑—up-regulation; ↓—down-regulation.

**Table 2:** Apoptosis inducing marine sponge.

Classification	Compound/ Extract	Source	Cell Line/Animal Model	Dose; Duration	Efficacy	Mechanism	Reference
Marine sponge	Leiodermatolide	*Leiodermatium*	AsPC-1, BxPC-3, MIA PaCa-2, PANC-1	10 nM; 24 h	Induction of apoptosis	↑c-caspase-3	[27]
			L3.6pl cells bearing mice	10 mg/kg; 3 weeks	Reduction of tumor weight		

↑—up-regulation.

**Table 4:** Metastasis inhibiting natural products.

Classification	Compound/Extract	Source	Cell Line/Animal Model	Dose; Duration	Efficacy	Mechanism	Reference
Fungus	Polyporenic acid	*Poria cocos*	PANC-1	30, 60 μM; 24 h	Inhibition of metastasis	↓CDC20	[68]
Fungus	*Poria cocos* EtOH extract			30, 60 μg/mL; 24 h			
Fungus	Terphenyllin		PANC-1, HPAC	25 μM; 24 h	Inhibition of invasion and migration		[69]
			SCID mice bearing PANC1 orthotopic tumors	20 mg/kg/day; 5 weeks	Inhibition of metastasis		
Plant	Cordifoliketones A	*Codonopsis cordifolioidea*	AsPC-1, BxPC-3, PANC-1	2, 4, 6 μg/mL; 12 h	Inhibition of invasion and migration		[54]
Plant	*Hormophysa triquetra* polyphenol	*Hormophysa triquertra*	PANC-1, PANC-3.27, BxPC-3, MIA PaCa-2	100 µg/mL; 24 h	Inhibition of resistant cell migration/invasion	↓CXCR4, COX-2, β-catenin, MMP-9, Ki-67, BAPX, PhPT-1, MEGF10	[70]
			MIA PaCa-2 bearing NCr-nu/nu nude mice	10 mg/kg; 3 weeks			
Plant	*Moringa oleifera* leaves water extract	Leaves of *Moringa oleifera*	PANC-1	0.4, 0.8, 1.8 mg/mL; 24 h, 2, 4 Gy radiation	Inhibition of metastasis		[59]
Plant	Sennoside A	*Rheum rhabarbarum*	MIA PaCa-2, PANC-1	10 μmol/L; 24 h	Inhibition of invadopodia formation	↑p-cofilin	[71]
			PANC-1	10 μM; 20 m	Inhibition of invasion and migration		
			PANC-1-Luc bearing BALB/c nu/nu mice	10 mg/kg; 10 days	Inhibition of metastasis		
Plant	Toosendanin		AsPC-1, PANC-1	50, 100, 200 nM; 24 h	Inhibition of invasion and migration	↑E-cadherin ↓Vimentin, ZEB1, Snail, p-AKT, p-PRAS40, p-mTOR, p-p70S6K	[72]
			PANC-1 bearing BALB/c mice	0.2 mg/kg; 28 days	Inhibition of EMT	↑E-cadherin ↓Vimentin, ZEB1, Snail	

CDC20, cell division cycle protein 20; CXCR-4, C-X-C chemokine receptor type 4; COX-2, cyclooxygenase-2; MMP-9, matrix metallopreteinase-9; BAPX, bagpipe homeobox homolog; PhPT-1, phosphohistidine phosphatase-1; MEGF10, multiple EGF-like domains 10; EMT, epithelial mesenchymal transition; ZEB1, zinc finger E-box-binding homeobox 1; PRAS, proline rich protein; mTOR, mammalian target of rapamycin; ↑, up-regulation; ↓, down-regulation.

**Table 5:** Angiogenesis inhibiting natural products.

Classification	Compound/Extract	Source	Cell Line/Animal Model	Dose; Duration	Efficacy	Mechanism	Reference
Plant	Danggui-Sayuk-Ga-Osuyu-Saenggang-Tang (DSGOST)	*Angelica gigas,Cinnamomum cassia* Blume, *Paeonia lactiflora* Pallas, *Akebia quinata* var. polyphylla Nak., *Asarum sieboldii* var. seoulense Nakai, *Glycyrrhiza uralensis* Fischer, *Zizyphus jujuba var. inermis* Rehder, *Evodia* rutaecarpa var. bodinieri Huang, *Zingiber officinale* Rosc.	HUVECs, HDMECs	100 µg/mL; 72 h	Inhibition of migration Inhibition of tube formation	↓p-VEGFR2, p-FAK, p-SRC, p-AKT, p-IKKα/β, p-IκBα, p-NF-κB, MMP-9	[73]
			PANC-28 bearing BALB/c nude mice	20 mg/kg; 49 days	Inhibition of angiogenesis	↑c-caspase-3 ↓Ki-67, p-VEGFR2, MMP-9	
				100 µg; 0.5 h			
Plant	SH003	*Astragalus membranaceus, Angelica gigas, Trichosanthes Kirilowii* Maximowicz	HUVECs	10, 20, 50 µg/mL; 24 h	Inhibition of angiogenesis	↑c-caspase-3 ↓p-VEGFR2, MMP-9, p-FAK, p-SRC, p-ERK, p-AKT, p-STAT3	[74]
			PANC-28 bearing BALB/c nude mice	2 mg/kg; 49 days		↑c-caspase-3 ↓Ki-67, p-VEGFR2, MMP-9	
				20 µg; 0.5 h			

HUVECs, human umbilical vascular endothelial cells; HDMECs, human dermal microvascular endothelial cells; p-VEGFR2, phosphorylated vascular endothelial growth factor 2; p-FAK, phosphorylated focal adhesion kinase; p-AKT, phosphorylated protein kinase B; p-IKKα/β, phosphorylated inhibitor of nuclear factor kappa-B kinaseα/β; p-NF-κB, phosphorylated nuclear factor kappa B; MMP-9, matrix metallopeptidase 9; p-ERK, phosphorylated extracellular signal-regulated kinase; p-STAT3, phosphorylated signal transducers and activators of transcription 3; ↑—up-regulation; ↓—down-regulation.

**Table 6:** Resistance inhibiting natural products.

Classification	Compound/Extract	Source	Cell Line/Animal Model	Dose; Duration	Efficacy	Mechanism	Reference
Plant	Terpinen-4-ol		COLO357, PANC-1, MIA-PaCa	0.005, 0.01, 0.05, 0.1%; 72 h	Inhibition of tumor growth Sensitization of gemcitabine		[80]
Animal	Scalarin	*Euryspongia* cf. *rosea*	PANC-1, MIA PaCa-2	10 µg/mL; 24 h	Inhibition of autophagy	↓RAGE	[81]
Plant	Bitter melon juice	*Momordica charantia*	AsPC-1	1–4%; 24, 48 h	Inhibition of viability	↓p-AKT, p-ERK1/2, p-PI3K, p-PTEN	[82]
Plant	Coix seed emulsion	*Coix lac* *h* *ryma-jobi*	PANC-1	4.0 mg/mL; 72 h	Sensitization of gemcitabine	↑caspase-3, c-PARP, Bax ↓NF-κB, Bcl-2, survivin, COX-2	[53]
			BxPC-3 bearing BALB/c nude mice	12.5 mL/kg; 24 days			
Plant	Coix seed extract	*Coix lac* *h* *ryma-jobi*	BxPC-3, PANC-1	10 mg/mL; 24, 48 h	Sensitization of gemcitabine	↓ABCB1, ABCG2	[76]
			BxPC-3 bearing BALB/c nude mice	12.5 mL/kg; 3 weeks			
Plant	Enzyme-treated asparagus extract		KLM1-R	2 mg/mL; 120 h	Sensitization of gemcitabine	↓HSP27, p-HSP27	[77]
Plant	EriB/ethanol extract	*Isodon eriocalyx*	SW1990	2.5 µM; 24 h	Sensitization of gemcitabine	↑c-caspase 3, c-PARP, p-JNK ↓p-PDK1, p-AKT1	[79]
Plant	Oat bran ethanol extract	*Avena sativa* L.	PANC-1, MIA PaCa-2	40 µg/mL; 72 h	Sensitization of gemcitabine	↑p-AMPK, p21, p27 ↓p-JNK, cyclin D1, CDk4, RRM1, RRM2	[78]
Plant	Pao Pereira extract	*Geissospermum vellosii*	PANC-1, MIA PaCa-2	50, 100 µg/mL; 48 h	Inhibition of tumor sphenoid formation Reduction of pancreatic CSCs	↓ CD44, CD24, EpCam, Nanog, β-catenin, BCL2L2, COX-2	[83]
			PANC-1 bearing nude mice.	20 mg/kg; 3 weeks	Reduction of pancreatic CSCs		
Plant	Qingyihuaji	Herba *Scutellariae barbatae*, Herba *Hedyotdis*, Rhizoma *Arisaematis erubescentis,* Herba seu Radix *Gynostemmatis pentaphylli*, Fructus Amomi Rotundus	CFPAC-1	40 μg/L; 24, 48, 72h	Sensitization of gemcitabine Inhibition of proliferation Decrease of migration	↑lncRNA AB209630 ↓miR-373, EphB2, Nanog	[84]
			CFPAC-1 bearing nude mice	40 g/kg; 28 days	Sensitization of gemcitabine Inhibition of proliferation		
Plant	*Rauwolfia vomitoria* root extract	*Rauwolfia vomitoria*	PANC-1, MIA PaCa-2	50, 100, 200 µg/mL; 48 h	Inhibition of tumor spheroid formation Reduction of pancreatic CSCs	↓CD24, EpCam, Nanog, β-catenin	[85]
			PANC-1 bearing athymic NCr-nu/numice	20 mg/kg; 5 week	Reduction of tumorigenicity		

CSCs, pancreatic cancer stem-like cells; RAGE, receptor for advanced glycation end products; c-PARP, cleaved-poly ADP ribose polymerase; Bax, Bcl-2-associated X protein; NF-κB, nuclear factor kappa B; Bcl-2, B-cell lymphoma-2; COX-2, cyclooxygenase-2; ABCB1, ATP-binding cassette B1; ABCG2, ATP-binding cassette G2; HSP27, heat-shock protein 27; p-JNK, phosphorylated c-Jun N-terminal kinase; PDK1, pyruvate dehydrogenase kinase 1; AKT1, protein kinase B1; CDk4, cyclin-dependent kinase 4; RRM1, ribonucleotide reductase subunit M1; RRM2, ribonucleotide reductase subunit M2; EpCam+, epithelial cell adhesion molecule+; ↑—up-regulation; ↓—down-regulation.

## Data Availability

Not applicable.

## Abbreviations

c-caspase	cleaved caspase
c-PARP	cleaved poly adenosine diphosphate ribose polymerase
Bax	Bcl-2-associated X protein
Bcl-family	B cell lymphoma-family
miRNA	microRNA
BRCA1/2	breast cancer susceptibility gene 1/2
PALB2	partner and localizer of BRCA2
TCM	Traditional Chinese Medicine
TTM	Traditional Thai Medicine
TKM	Tracitional Korean Medicine
CAM	Complementary and Alternative Medicine
PCD	programmed cell death
AbE	*Agaricus blazei* Murrill water extract
DAPK3	death-associated protein kinase 3
NLRP1	NACHT, LRR, and PYD domains-containing protein 1
TRAIL	tumor necrosis factor-related apoptosis inducing ligand
ROS	reactive oxygen species
MMP	mitochondrial membrane potential
compound 17	14-deoxy-11,12-didehydroandrographolide
DMC	2′,4′-Dihydroxy-6′-methoxy-3′,5′-dimethylchalcone
flavone A	5,7-dihydroxy-3,6,8-trimethoxyflavone
flavone B	3,5-dihydroxy-6,7,8-trimethoxyflavone
PCNA	proliferating cell nuclear antigen
EGFR	epidermal growth factor receptor
AKT	protein kinase B (PKB)
EZH2	enhancer of Zeste homolog 2
ECH	Echinacoside
MAPK	mitogen-activated protein kinase
GSPs	grape seed proanthocyanidins
CDK6	cell division protein kinase 6
MSH6	MutS homolog 6
DNMT1	DNA methyltransferase 1
JNK	c-Jun N-terminal kinase
c-Bid	cleaved-Bcl-2 homology 3 interacting domain death agonist
GSH	Glutathione
MGR	mastic gum resin
MGDG	monogalactosyl diacylglycerol
XIAP	X-linked inhibitor of apoptosis
COX-2	cyclooxygenase 2
VEGF	vascular endothelial growth factor
MMP-9	matrix metalloproteinase 9
NF-κB	nuclear factor kappa B
RN1	a polysaccharide from the flower of *Panax notoginseng*
Gal-3	Galectin-3
ERK	extracellular signal-regulated kinase
Runx1	Runt-related transcription factor 1
Skp2	S-phase kinase associated protein 2
WA	Withaferin A
CA	Carnosol
HGF	hepatocyte growth factor
c-Met	mesenchymal-epithelial transition factor
BAEE	Bitter apricot ethanolic extract
SN	non-polar stem extracts
cIAP-2	cellular inhibitor of apoptosis 2
CSE	coix seed emulsion
F1	*Eucalyptus microcorys* aqueous extract
H3	herbal mixture ethanol extract
CXCR-4	C-X-C motif chemokine receptor 4
JAK2	Janus kinase 2
RBC	red blood cell
EEIHL	ethyl acetate extract of *Inula helenium* L.
STAT3	signal transducers and activators of transcription 3
PS	*Paeonia suffruticosa* aqueous extracts
SSBE	the extract of *Sedum sarmentosum Bunge*
TFAE	total flavonoid aglycones extract
F35	alloalantolactone, alantolacton, isoalantolactone [1:5:4]
PaEBE	*Pterospermum acerifolium* ethanolic bark extract
p-c-JUN	phospho-c-Jun
pS6	phospho-S6
DCLK-1	doublecortin-like kinase 1
Shh	sonic hedgehog signaling molecule
PTEN	phosphatase and tensin homolog
E-cadherin	epithelial cadherin
Mcl-1	myeloid cell leukemia 1
EMT	epithelial mesenchymal transition
CDC20	cell division cycle protein 20
CXCR-4	C-X-C chemokine receptor type 4
HT-EA	*Hormophysa triquertra* polyphenol
SSH	slingshot homologs
ZEB	zinc finger E-box-binding homeobox
mTOR	mammalian target of rapamycin
PRAS	proline rich protein
BAPX	bagpipe homeobox homolog
PhPT-1	phosphohistidine phosphatase 1
MEGF10	multiple EGF-like domains 10
DSGOST	Danggui-Sayuk-Ga-Osuyu-Saenggang-Tang
HUVECs	human umbilical vascular endothelial cells
HDMECs	human dermal microvascular endothelial cells
VEGFR2	vascular endothelial growth factor 2
p-FAK	phosphorylated focal adhesion kinase
p-IKKα/β	phosphorylated inhibitor of nuclear factor kappa-B kinaseα/β
RAGE	receptor for advanced glycation end products
ABC	ATP-binding cassette
ABCB1	ATP-binding cassette B1
ABCG2	ATP-binding cassette G2
ETAS	enzyme-treated asparagus extract
HSP	heat-shock protein
EriB	Eriocalyxin B
PDK1	pyruvate dehydrogenase kinase 1
OBE	Oat bran ethanol extract
RRM1	ribonucleotide reductase subunit M1
RRM2	ribonucleotide reductase subunit M2
AMPK	AMP-activated protein kinase
CSCs	cancer stem-like cells
EpCam+	epithelial cell adhesion molecule+
BCL2L2	Bcl-2 like protein 2
lncRNA	long non-coding RNAs
HPNE	human pancreatic epithelial nestin-expressing
CDk4	cyclin-dependent kinase 4
DLTs	dose-limiting toxicities
IQuS	Iscador Qu Spezial
